# Disparities in spatially variable gene calling highlight the need for benchmarking spatial transcriptomics methods

**DOI:** 10.1186/s13059-023-03045-1

**Published:** 2023-09-18

**Authors:** Natalie Charitakis, Agus Salim, Adam T. Piers, Kevin I. Watt, Enzo R. Porrello, David A. Elliott, Mirana Ramialison

**Affiliations:** 1 Murdoch Children’s Research Institute, Royal Children’s Hospital, Flemington Road, Parkville, VIC 3052 Australia; 2 Department of Paediatrics, University of Melbourne, Grattan Street, Parkville, VIC 3010 Australia; 3 Novo Nordisk Foundation Center for Stem Cell Medicine, Murdoch Children’s Research Institute, Royal Children’s Hospital, Flemington Road, Parkville, VIC 3052 Australia; 4 Melbourne School of Population and Global Health, University of Melbourne, Bouverie St, Carlton, VIC 3053 Australia; 5 School of Mathematics and Statistics, University of Melbourne, Swanston Street, Parkville, VIC 3010 Australia; 6 Melbourne Centre for Cardiovascular Genomics and Regenerative Medicine, The Royal Children’s Hospital, Melbourne, VIC 3052 Australia; 7 Department of Anatomy and Physiology, University of Melbourne, Grattan Street, Parkville, VIC 3010 Australia; 8 Department of Diabetes, Monash University, Alfred Centre, Commercial Road, Melbourne, VIC 3004 Australia

## Abstract

Identifying spatially variable genes (SVGs) is a key step in the analysis of spatially resolved transcriptomics data. SVGs provide biological insights by defining transcriptomic differences within tissues, which was previously unachievable using RNA-sequencing technologies. However, the increasing number of published tools designed to define SVG sets currently lack benchmarking methods to accurately assess performance. This study compares results of 6 purpose-built packages for SVG identification across 9 public and 5 simulated datasets and highlights discrepancies between results. Additional tools for generation of simulated data and development of benchmarking methods are required to improve methods for identifying SVGs.

**Supplementary Information:**

The online version contains supplementary material available at 10.1186/s13059-023-03045-1.

## Background

Spatially resolved transcriptomics (SRT) captures variations in gene expression across tissues [1–7]. Computational methods for analysis of SRT data are being established [8–12]. Among the goals of SRT analysis pipelines is the robust and reliable identification of spatially variable genes (SVGs) within tissue sections [12–14]. SVGs are defined as having expression levels across a tissue that covary in a location specific manner [13, 15]. Published methods for SVG identification employ different mathematical models aiming to capture biological truth [13, 14, 16–27]. Benchmarking of analysis tools is needed to ensure the reliability of processed data matches or supersedes that of similar technologies such as single-cell (sc) RNA-Seq [28, 29].

Here, we compared the performance of six packages—SpatialDE [13], SPARK-X [16], Squidpy [30], Seurat [18], SpaGCN [17], scGCO [27] from healthy and cancerous fresh frozen (FF) and formalin-fixed paraffin embedded (FFPE) tissues generated with 10X Visium technology alongside simulated datasets (Additional file 1: Fig. S1) [31, 32]. Our analysis reveals discrepancies in SVGs identified by each package highlighting the urgent need for further benchmarking and development of tools to identify SVGs.

## Results and discussion

**Fig. 1:**
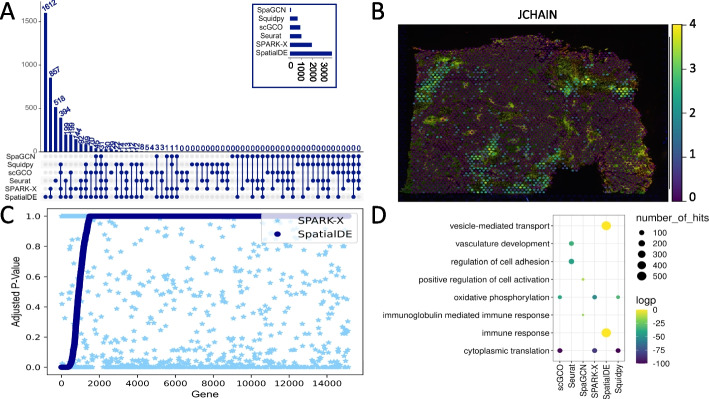
Discrepancies between SVGs in a dataset annotated by six different packages. **A** Upset plots displaying the distinct intersections of SVGs identified in FF endometrial adenocarcinoma ovarian tissue dataset when analysed with SpaGCN, Squidpy, scGCO, Seurat, SPARK-X and SpatialDE. Bar chart in box displays the total number of SVGs identified by each package. **B** Pattern of expression of *JCHAIN*, a SVG identified by all six packages in FF endometrial adenocarcinoma ovarian tissue. Spots representing the capture regions of the Visium slide are overlayed on accompanying histological image. **C** Sorted *q*-values from SpatialDE results across the FF left ventricle datasets plotted against SPARK-X *q*-values for the same gene. **D** Gene ontology enrichment results from FF endometrial adenocarcinoma ovarian tissue dataset using SVGs identified by each package as inputs

Comparing SVGs predicted by six packages, we found that the numbers of SVGs identified differed by one to two orders of magnitude across the same tissue for all nine investigated datasets (Fig. 1A and Additional file 1: Fig. S2). For example, in the FF endometrial adenocarcinoma ovarian tissue dataset, the number of predicted SVGs ranged from 87 to 3707 (Fig. 1A). For 7/9 datasets, SpatialDE identifies the greatest number of SVGs, while SpaGCN identifies the fewest number of SVGs in 6/9 public datasets (Fig. 1A and Additional file 1: Fig. S2). Apart from the FF mouse brain coronal section dataset, packages tested tend to report more SVGs in cancerous datasets than in healthy tissues, most evident across SpatialDE and SPARK-X (Additional file 1: Fig. S3). There is no apparent difference in reported SVGs driven by data generated from FF or FFPE tissue (Additional file 1: Fig. S3).

To understand the disparity in the number of SVGs reported, we hypothesised that methods used to correct for type I error might influence results. SpatialDE, Squidpy, scGCO and SPARK-X report a *q*-value/FDR for all genes. Methods that do not supply associated *p*-values report the lowest number of SVGs in half of the datasets (Fig. 1A and Additional file 1: Fig. S2). Across all datasets, a minimal number of common SVGs are identified between all 6 packages that each display an expression pattern that aligns with spatial correlation suggesting that they are bona fide SVGs (Fig. 1B).

Next, to assess the significance of small overlap between predicted SVGs, the ranked lists of SVGs provided by SpatialDE, Squidpy, scGCO and SPARK-X were compared across datasets using a Wilcoxon signed rank test. We observed differences in the SVG ranking across the tested packages, indicating that the lack of overlap is statistically significant (Fig. 1C and Additional file 1: Fig. S4-9, Additional file 2: Table S1). *q*-values for a single gene differ between packages (Fig. 1C and Additional file 1: Fig. S4-9).

We investigated the downstream impact arising from discordant results by comparing gene ontology (GO) enrichment analysis using SVGs predicted by each package within the same tissue (Fig. 1D and Additional file 1: Fig. S10). We observed non-overlapping parent terms between datasets in the top 10 over-represented GO terms (Fig. 1D and Additional file 1: Fig. S10). These results suggest that gene sets predicted by each package do not overlap functionally. Further, restricting GO analysis to SVGs can bias downstream interpretation of results, especially if there is a risk of a large percentage of false positives (FP).

**Fig. 2:**
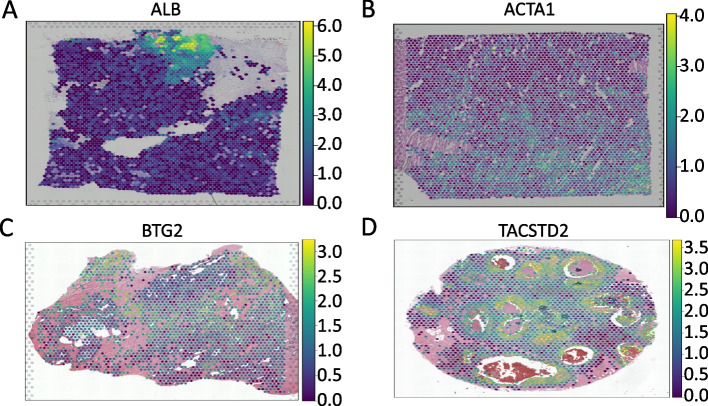
Expression patterns of SVGs identified by all tested packages in a single dataset. **A** Pattern of expression of *ALB*, a SVG identified by all six packages in FF invasive ductal carcinoma breast tissue. **B** Pattern of expression of *ACTA1*, a SVG identified by all six packages in FF left ventricle. **C** Pattern of expression of *BTG2*, a SVG identified by all six packages in FFPE prostate. **D** Pattern of expression of *TACSTD2*, a SVG identified by all six packages in FFPE invasive ductal carcinoma breast tissue

Despite the discrepancies, SVGs identified by all packages within the same dataset display an expression pattern aligned with a clear spatial correlation (Fig. 2). An example is *JCHAIN*, which is expressed in endometrial macrophages and associated with adenocarcinoma (Fig. 1B) [33–35]. Furthermore, in FF mouse brain coronal section, a dataset with known spatial gene expression patterns, many of the 368 commonly identified SVGs display expression patterns corresponding to their known region of expression in the Allen Mouse Brain Atlas (Additional file 1: Fig. S11) [36]. These observations indicate that patterns with a stronger signal-to-noise ratio are identified by all methods.

To gain insight into the inconsistent results between different packages we evaluated their sensitivity and specificity. To determine true positives and FP rates, we first generated two simulated datasets with different known SVG patterns with SRTsim (Additional file 1: Fig. S12 A-B) (see Methods) [37]. Each dataset contained 1500 known SVGs (see methods). Using each as the input to four packages (scGCO, SpatialDE, Squidpy and SPARK-X), all packages identify all true positives across datasets, except for Squidpy when analysing the dataset with lower signal SVGs. This indicates a potential need for a more relaxed Moran’s *I* statistic threshold (Additional file 2: Table S2). Additionally, most packages introduce minimal levels of FPs with simulated datasets, except for scGCO (Additional file 2: Table S2). This is possibly due to of the type I error correction method or sequential search procedure employed by scGCO [27].

To further investigate the potential of each package to call FPs, we generated four negative control datasets from two publicly available datasets. For each, two versions of negative controls were generated—[1] where spatial coordinates are permutated and [2] where spatial coordinates and columns of gene expression matrix are permutated. For all negative control datasets generated from the FF left ventricle, Seurat, scGCO, Squidpy and SPARK-X identified no SVGs, indicating an ability to distinguish signal from noise. SpaGCN identified < 10 SVGs. SpatialDE identified 4 SVGs when both spatial coordinates and columns of the gene expression matrix are permutated and 2953 in the other dataset. For negative control datasets generated from FFPE prostate randomising only spatial coordinates, SpatialDE identified 632 SVGs, SpaGCN identified 232 and Squidpy identified 1. Seurat, scGCO and SPARK-X did not run with these inputs. When spatial coordinates and gene expression values were randomised, SpatialDE was the only package to identify SVGs (713). Patterns of the top SVGs identified by SpatialDE lack a strong spatial pattern (Additional file 1: Fig. S13). This would indicate that SpatialDE is prone to introducing FPs into a dataset when genes are lowly expressed compared. However, the original SVGs identified by SpatialDE and the result in Fig. 1B suggest the top ranked SVGs labelled by SpatialDE in real data display a signal consistent with that of a SVG.

Inconsistencies in the results between different packages may therefore be explained by the differences in FP rates, rather than the rate of false negatives. To investigate this, expression of known housekeeping gene *Eef2* [38] was visualised across the FF mouse brain coronal Section dataset to verify its ubiquitous expression (Additional file 1: Fig. S11 D). *Eef2* was found to be annotated as an SVG by scGCO, SPARK-X, SpatialDE and Squidpy. This indicates genes with high levels of expression, but that do not display strong spatial expression patterns, may be a source of confounding.

In addition to differences in FP rates, results generated from different packages applied to the same dataset may arise due to the different assumptions regarding the underlying distributions of gene expression and effectiveness of normalisation methods (Additional file 2: Table S3). Negative binomial distributions can successfully model sequencing-based methods that employ unique molecular identifiers (UMIs) and most gene expression patterns in Visium datasets [37, 39]. While certain packages implement their own normalisation method, there is no guarantee that data pre-processed using a separate workflow will have been normalised to fit the assumptions of gene distributions held by a particular package [13, 18]. Once methods for generating simulated SRT datasets with different underlying distributions of gene expression are published, the effect this has on identifying SVGs needs to be investigated thoroughly. There are many caveats to generating simulated datasets with known patterns of gene expression with efforts focused on simulating data with defined cell types [40–47].

## Conclusions

While different mathematical models for detecting SVGs produce varied results when applied to the same tissue, and across tissues and biological conditions, SVGs with a strong spatial covariance are consistently identified. This indicates that differences between methods do not bias performance in a particular tissue or biological condition. However, more transcriptionally heterogenous tissues appear to affect the performance of certain packages in calling SVGs and genes with unclear spatial patterns but high expression levels may be labelled as FP [14, 15]. Only four of the tested packages produced *q*-value ranked gene lists; thus, differences in FP rates could confound comparisons of results across packages. Furthermore, due to the resolution of the Visium technology platform, the presence of heterogenous cell types could complicate accurate identification of SVGs due to inherent transcriptional variation. Increased resolution may present challenges in terms of computational performance and the mathematical difficulties of dealing with highly sparse datasets. As SVG identification becomes an integral part of the computational analysis of SRT and datasets grow, a subset of SVGs could be used in preference over the entire dataset for downstream analysis (such as highly variable genes in scRNA-Seq analysis). Introducing noise may hamper key downstream steps in understanding spatially restricted disease states as well as novel targets for treatment [48, 49]. Methods that explore the entire dataset for SVG labelling paired with stricter *q*-value cut-offs (*q*-value < 0.01) can be employed when analysing tissues with known transcriptional complexity to decrease the possibility of FP being reported. Future work will include comparison of package performance across datasets generated from different SRT platforms. The development of workflows for simulating datasets and benchmarking current and novel methods for SVG identification will allow for accurate determination of the spatially restricted transcriptional differences that manifest as biological outcomes during development and disease.

## Methods

The aim of this study is to compare publicly available tools that were developed to identify SVGs within spatially resolved transcriptomics (SRT) datasets. When individual tools are published their performance is reported across SRT datasets generated using various platforms, but a systematic comparison of package performance to identify SVGs in FF and FFPE preserved healthy and diseased tissues is lacking. Here, we elected to focus on comparing package performance on data generated using the 10X Visium platform, due to the early commercial availability of the Visium platform which enables the generation of multiple datasets from different tissues and fixation protocols. The scripts generated for this analysis are available on Github [50] and Zenodo [51], with simulated datasets available through Zenodo.

Six state-of-the-art packages built for the identification of SVGs (Seurat and Squidpy excepted) in SRT data were selected for benchmarking: SpatialDE [13], SPARK-X [16], Seurat [18], SpaGCN [17], Squidpy [30] and scGCO [27]. Each package uses a different algorithm to identify SVGs and holds varied assumptions on the distribution of gene expression data (Additional File 2: Table S3). Packages were purposefully chosen to compass a variety of algorithms and associated mathematical models of gene distribution expression that can be used to identify these spatial patterns. While two packages used algorithms based on graph theory, the others were selected to test different mathematical assumptions regarding SRT data. SpatialDE employs Gaussian process regression, a non-parametric probabilistic model (Additional File 2: Table S3) [13]. SPARK-X is another non-parametric method, building on a covariance test framework, specifically the projection covariance function [16]. This function can measure similarity between two locations or gene expression, it can quantify if the product of the two inputs deviates significantly from the mean value [16]. Seurat employs a mark point process, first used by the Trendsceek package [52, 53]. This is another non-parametric approach that can test if gene expression levels are significantly dependent on the spatial distribution of spots as a function of the distance between them [52]. This can then calculate the mark-variogram [52, 53]. SpaGCN’s method is built around a graph convolutional network (GCN)-based approach and is unique in that it incorporates signal from histology images and restricts identification of SVGs to within spatial domains [17]. Gene expression and image data are converted into a weighted undirected graph, which then calculates the distances between any two vertices in the graph [17]. The nodes represent spots, and the edge weight is calculated using the histology image and the Euclidian distance between two vertices [17]. The weight of each edge is calculated as a function between how related spots are in the graph [17]. Dimensionality reduction then graph convolution is computed to then identify spatial domains [17]. Finally, differential expression analysis is performed between spots in one spatial domain with neighbouring domains utilising a Wilcoxon rank-sum test and genes with an adjust *p*-value < 0.05 are reported as SVGs [17]. scGCO is another graph-based method, but this package utilises a probabilistic graph model by optimising hidden Markov random fields (HMRF) for the purpose of SVG identification [27]. Similar to Seurat, scGCO uses a marked point process to model spatial gene expression [27, 53]. The dependency of gene expression states on spatial locations is analysed using the complete spatial randomness framework, and scGCO overcomes its limitations using HRMF [27]. Finally, Squidpy employs the spatial autocorrelation metrics of Moran’s *I* to label SVGs [30]. Given a continuous feature, in this case gene expression level and their spatial location, it can evaluate whether a pattern is present or not using Moran’s *I* [30].

Each package was tested on nine publicly available, V1 Chemistry Visium datasets generated from healthy and cancerous human tissues, along with a single mouse dataset (Additional File 2: Table S4). Additional testing was performed using simulated data (Additional File 1: Fig. S1). The filtered output files and imaging data from the Space Ranger v1.0.0 pipeline were downloaded for each dataset. Further filtering and pre-processing of the data was performed using Scanpy v1.8.1, and the following were removed: genes expressed in fewer than 10 spots, spots with fewer than 2000 counts, and/or spots with fewer than 2000 genes expressed. As the percentage of mitochondrial gene expression and maximum number of counts per spot varied significantly between datasets, filtering was performed on a per-dataset basis. Mitochondrial filtering in FF Cerebellum was limited to spots with less than 15% mitochondrial genes [54]. Counts were then normalised per spot, log transformed and the top 2000 highly variable genes identified. This count matrix was used as the input files for each algorithm. A custom script was written for conversion between the Scanpy AnnData object and the Seurat S4 object input.

Analysis using SpatialDE was performed using default parameters using Python v 3.9.7. Reported SVGs were filtered to include only those with an *q*-value <  = 0.05.

Analysis using SPARK-X v 1.1.1 was performed with default parameters using R v 3.6.1. Reported SVGs were filtered to include only those with an adjusted *p*-value <  = 0.05.

Analysis using Seurat v 4.1.0 was performed with default parameters using R v 3.6.1. A custom script was used to convert the Anndata to Seurat object. SVGs were identified using the function FindSpatiallyVariableFeatures with default parameters as specified in the vignette (top 1000 variable features selected and markvariogram selection method).

Analysis using SpaGCN v 1.2.0 using default parameters after calculating the appropriate radius for each dataset using Python v 3.9.7. Number of target domains was informed by Scanpy clustering, and SVGs that were common between domains were removed.

Analysis using scGCO v 1.1.1 with default parameters using Python v 3.7.6. Reported SVGs were filtered to include only those with an FDR value <  = 0.05.

Comparisons of the number of SVGs identified by each package across all datasets visualised in Fig. S3 (Additional File 1) were modified from code generated by ChatGPT [55].

Upset plots were generated using package upsetplot v 0.6.0 and Python v 3.9.7. Pattern of SVG expression was generated using Scanpy v1.8.1. The upset plots display the unique intersection of different overlapping or independent results identified across packages within a single dataset and serve to highlight the differences in the scale of the number of SVGs identified within a dataset. Furthermore, visualisation of datasets with the most reported SVGs independent of the other packages may highlight the introduction of FP.

The SVGs identified by each package were used as the inputs for gene ontology enrichment analysis. When less than 3000 SVGs were identified, Metascape was used for analysis, while in instances where there were over 3000 inputs, WebGestalt was used as Metascape has a limit on the number of genes accepted as inputs [56, 57]. Metascape was run with express analysis and the input as species was set to *H.sapiens* or *M.musculus*. For WebGestalt analysis, the organism of interest was set as Homo sapiens or Mus musculus, method of interest as over-representation analysis, functional database of ‘gene ontology’ focusing on biological process and the ‘reference set’ was set as ‘genome’. All results were downloaded then filtered down to the top 10 significant GO terms. ggplot2 package was used to visualise GO terms.

Seurat ranks highly variable genes by how dependent their expression is on spatial location, then produces a list of SVGs [53]. SpaGCN reports unraked lists of SVGs with a *p*-value of < 0.05. For the four packages that generated *p*-values associated with the identified SVGs (SpatialDE, Squidpy, SPARK-X and scGCO), a Wilcoxon signed rank test was performed. For each of the nine publicly available datasets, each possible pairwise combination of the results from different packages was analysed using the scipy stats function to perform a Wilcoxon signed rank test. The concordance for each dataset was calculated using a pairwise comparison between the results of SpatialDE, Squidpy, SPARK-X and scGCO for all available combinations.

Simulated datasets with known patterns of SVGs were generated using SRTsim [37]. SRTsim allows the user to generate reference-free, simulated data that captures many of the features typical of Visium datasets through a Shiny app interface [37]. These features include the number of spots and genes in the dataset and the mean and dispersion values of gene expression as well as the proportion of zero counts [37]. If regions are selected within the dataset, the log fold-change within this region can be set compared to the rest of simulated area. Expression counts were simulated using a negative binomial distribution for both datasets [37]. Two datasets were generated using SRTsim for this purpose. A square grid is selected (reproducible seed 2), with 4000 spots and 15,000 genes overall. A square ‘hotspot’ pattern of 675 spots was generated in the centre of the grid, which will serve the area of higher gene expression for the simulated SVGs (Additional File 1: Fig. S12 A). One thousand five hundred signal genes were then simulated with 13,500 noise genes; no lower signal genes were selected. A reproducible seed of 2 was set, and baseline overdispersion was set to 0.5 with the baseline mean set to 3. A second simulated dataset was generated with SRTsim with a slightly more complex gene expression pattern. The grid layout and number of spots was the same as the aforementioned dataset, set with reproducible seed 3. Here, two separate regions were generated to contain simulated SVG expression, in opposite corners of the square layout (Additional File 1: Fig. S12 B). The two groups had a log fold-change of 5 compared to the rest of the area (group A) with a log fold-change of 1. Next, the expression of 15,000 genes were simulated across the dataset: 13,500 noise genes, 750 high signal SVGs and 750 low signal SVGs. Dispersion and mean values were the same as the previous dataset. This second dataset was generated with the aim of showcasing which packages may be adept at identifying SVGs with a lower signal across the dataset or a pattern different to the typical ‘hotspot’ which is often shown, such as in early simulations available with SPARK-X [16]. The overlap of the results from each package with the known SVGs and noise genes in the data was then calculated to determine the sensitivity and specificity of each package. R scripts to calculate the sensitivity and specificity of each package were modified from code generated by ChatGPT [55].

A total of four negative control datasets were generated, two by randomising the values of the FF left ventricle data and two by randomising the values of the FFPE prostate data in python. For each of the two datasets, one simulated dataset was created by randomising the x and y spot coordinates for this dataset independently to remove spatial correlation. Subsequently, an additional simulated dataset was generated from each dataset by randomising the spatial coordinates and each column in the gene expression matrix to ensure any overall associations were removed. To generate a simulated dataset of known coordinates, the code available in the SPARK-X simulated data vignette was used. To test all simulated datasets, the same parameters for each algorithm were used as in the analysis of the publicly available datasets. The true positives rates and false positive rates were then calculated for the results all packages that were compatible with the dataset.

### Supplementary Information

**Additional file 1: Fig. S1.** Overview of the design of benchmarking workflow. First, publicly available datasets are obtained to be used as inputs for pre-processing in a Scanpy workflow. For each dataset the processed output is then transformed into a data object most suitable for each of the SVG analysis packages. Simulated data is directly used as inputs for SVG analysis. Finally, the results are compared across packages within each dataset. **Fig S2.** Distinct overlap of SVGs identified by different combinations of the six tested packages. A) FF cerebellum. B) FF lymph node. C) FFPE adenocarcinoma prostate. D) FF invasive ductal carcinoma breast tissue. E) FF left ventricle. F) FFPE prostate. G) FFPE invasive ductal carcinoma breast tissue. H) FF mouse brain coronal section. **Fig S3.** Comparison of the number of SVGs identified by six different packages across different datasets. FFPE tissues are visualised by a cross and FF tissues are visualised with a square. High numbers of SVGs are identified in both FF and FFPE tissues, however tissues that are more transcriptionally complex seem to have more SVGs called across the dataset. Seurat reports a consistent number of SVGs across datasets as it first identifies highly variable genes then ranks their expression by how dependent it is on spatial location [53]. **Fig S4.** Comparison of ranked SpatialDE q-values against gene-matched SPARK-X q-values of all genes generated from each dataset. Order of plots repeats across all datasets. A) FF cerebellum. B) FF lymph node. C) FFPE adenocarcinoma prostate. D) FF invasive ductal carcinoma breast tissue. E) FFPE prostate. F) FFPE invasive ductal carcinoma breast tissue. G) FF endometrial adenocarcinoma ovarian tissue. H) FF mouse brain coronal section. **Fig S5.** Comparison of ranked SpatialDE q-values against gene-matched scGCO q-values of all genes generated from each dataset. A) FF cerebellum. B) FF lymph node. C) FFPE adenocarcinoma prostate. D) FF invasive ductal carcinoma breast tissue. E) FFPE prostate. F) FFPE invasive ductal carcinoma breast tissue. G) FF endometrial adenocarcinoma ovarian tissue. H) FF left ventricle. I) FF mouse brain coronal section. **Fig S6.** Comparison of ranked SPARK-X q-values against gene-matched scGCO q-values of all genes generated from each dataset. A) FF cerebellum. B) FF lymph node. C) FFPE adenocarcinoma prostate. D) FF invasive ductal carcinoma breast tissue. E) FFPE prostate. F) FFPE invasive ductal carcinoma breast tissue. G) FF endometrial adenocarcinoma ovarian tissue. H) FF left ventricle. I) FF mouse brain coronal section. **Fig S7.** Comparison of ranked SpatialDE q-values against gene-matched Squidpy q-values of all genes generated from each dataset. A) FF cerebellum. B) FF lymph node. C) FFPE adenocarcinoma prostate. D) FF invasive ductal carcinoma breast tissue. E) FFPE prostate. F) FFPE invasive ductal carcinoma breast tissue. G) FF endometrial adenocarcinoma ovarian tissue. H) FF left ventricle. I) FF mouse brain coronal section. **Fig S8.** Comparison of ranked SPARK-X q-values against gene-matched Squidpy q-values of all genes generated from each dataset. A) FF cerebellum. B) FF lymph node. C) FFPE adenocarcinoma prostate. D) FF invasive ductal carcinoma breast tissue. E) FFPE prostate. F) FFPE invasive ductal carcinoma breast tissue. G) FF endometrial adenocarcinoma ovarian tissue. H) FF left ventricle. I) FF mouse brain coronal section. **Fig S9.** Comparison of ranked scGCO q-values against gene-matched Squidpy q-values of all genes generated from each dataset. A) FF cerebellum. B) FF lymph node. C) FFPE adenocarcinoma prostate. D) FF invasive ductal carcinoma breast tissue. E) FFPE prostate. F) FFPE invasive ductal carcinoma breast tissue. G) FF endometrial adenocarcinoma ovarian tissue. H) FF left ventricle. I) FF mouse brain coronal section. **Fig S10.** Gene ontology enrichment results using SVGs identified by each package as inputs across datasets. A) FF cerebellum. B) FF lymph node. C) FFPE adenocarcinoma prostate. D) FF invasive ductal carcinoma breast tissue. E) FF left ventricle. F) FFPE prostate. G) FFPE invasive ductal carcinoma breast tissue. H) FF mouse brain coronal Section. **Fig S11.** Spatial expression patterns of SVGs identified by all packages across the FF mouse brain coronal section dataset. A) Expression of *Hap1* across the hypothalamus and amygdala, cross-referenced with the Allen Mouse Brain Reference. B) Expression of *Prkcd* localised to the thalamus, cross-referenced with the Allen Mouse Brain Reference. C) Expression of *Itpka*, with highest expression in the isocortex, hippocampal formation (HPF) and cortical subplate consistent with patterns displayed in the Allen Mouse Brain Reference. D) Expression of *Eef2*, a known housekeeping gene in mouse (39). **Fig S12.** Simulated datasets generated with SRT sim. A) Location of simulated SVGs with a hotspot pattern visualised in blue, while red area indicates expression of noise genes. B) Location of simulated SVGs in both blue and green corners, while red area indicates expression of noise genes. C) Distinct overlap of SVGs compared to the control SVG list identified by different combinations of the four tested packages. 1500 SVGs were present in this dataset. D) Distinct overlap of SVGs compared to the control SVG list identified by different combinations of the four tested packages. 750 high signal SVGs and 750 low signal SVGs were present in this dataset. **Fig S13.** Top SVG identified by SpatialDE in negative simulated datasets generated by A) Randomising coordinates of FF Left Ventricle dataset. B) Randomising counts and coordinates of FF left ventricle dataset. C) Randomising coordinates of FFPE prostate dataset. D) Randomising counts and coordinates of FFPE prostate dataset. **Fig S14.** Upset plot of results generated from running SPARK-X and SpatialDE on simulated data with known pattern of SVGs.

**Additional file 2: Table S1.** Reported statistic and associated *p*-value from each combination of pairwise comparison of results between SpatialDE, SPARK-X, scGCO and Squidpy when reported *p*-values were compared using the Wilcoxon signed rank test. **Table S2.** Sensitivity and specificity of Squidpy, SPARK-X, SpatialDE and scGCO when used to analyse both simulated datasets generated with SRTsim. Sensitivity and specificity are reported over a range of q-value cut-offs, from 0.01-1.0. **Table S3.** Packages included for identification of SVGs in benchmarking study. The packages are ordered by assumptions made on distribution of gene expression. The rows highlighted in blue indicate grouped packages using graph-based methods. **Table S4.** Overview of publicly available 10X Visium datasets to be included in benchmarking process. FF indicates tissues that are fresh frozen and FFPE indicates tissues that are formalin-fixed paraffin-embedded. The filtered output files and imaging data from the Space Ranger v1.0.0, v1.2.0 or V1.3.0 pipeline were downloaded for each dataset.

**Additional file 3.** Review history

## Data Availability

The input files for the Visium data used in this study are available from the 10X Genomics website. Additional file 2: Table S4 contains access links to the publicly available datasets used in this study. Examples of the data pre-processing and scripts used in the analysis to identify SVGs on publicly available datasets for each of the six packages along with generated simulated data can be found on Zenodo under the https://doi.org/10.5281/zenodo.8208131 [51]. The scripts used throughout the analysis are also available on Github at Ramialison-Lab/Disparities_in_SVG_calling under a GPL3.0 license [50].
