# Supplementary material for: Disparities in spatially variable gene calling highlight the need for benchmarking spatial transcriptomics methods
Source: Genome Biol. 2023 Sep 18;24:209. doi: 10.1186/s13059-023-03045-1 (PMC10506280; doi:10.1186/s13059-023-03045-1)
Supplement: Supplementary file 1 — Additional file 1: Fig. S1. Overview of the design of benchmarking workflow. First, publicly available datasets are obtained to be used as inputs for pre-processing in a Scanpy workflow. For each dataset the processed output is then transformed into a data object most suitable for each of the SVG analysis packages. Simulated data is directly used as inputs for SVG analysis. Finally, the results are compared across packages within each dataset. Fig S2. Distinct overlap of SVGs identified by different combinations of the six tested packages. A) FF cerebellum. B) FF lymph node. C) FFPE adenocarcinoma prostate. D) FF invasive ductal carcinoma breast tissue. E) FF left ventricle. F) FFPE prostate. G) FFPE invasive ductal carcinoma breast tissue. H) FF mouse brain coronal section. Fig S3. Comparison of the number of SVGs identified by six different packages across different datasets. FFPE tissues are visualised by a cross and FF tissues are visualised with a square. High numbers of SVGs are identified in both FF and FFPE tissues, however tissues that are more transcriptionally complex seem to have more SVGs called across the dataset. Seurat reports a consistent number of SVGs across datasets as it first identifies highly variable genes then ranks their expression by how dependent it is on spatial location [53]. Fig S4. Comparison of ranked SpatialDE q-values against gene-matched SPARK-X q-values of all genes generated from each dataset. Order of plots repeats across all datasets. A) FF cerebellum. B) FF lymph node. C) FFPE adenocarcinoma prostate. D) FF invasive ductal carcinoma breast tissue. E) FFPE prostate. F) FFPE invasive ductal carcinoma breast tissue. G) FF endometrial adenocarcinoma ovarian tissue. H) FF mouse brain coronal section. Fig S5. Comparison of ranked SpatialDE q-values against gene-matched scGCO q-values of all genes generated from each dataset. A) FF cerebellum. B) FF lymph node. C) FFPE adenocarcinoma prostate. D) FF invasive ductal carcinoma breast [file 13059_2023_3045_MOESM1_ESM.pdf]

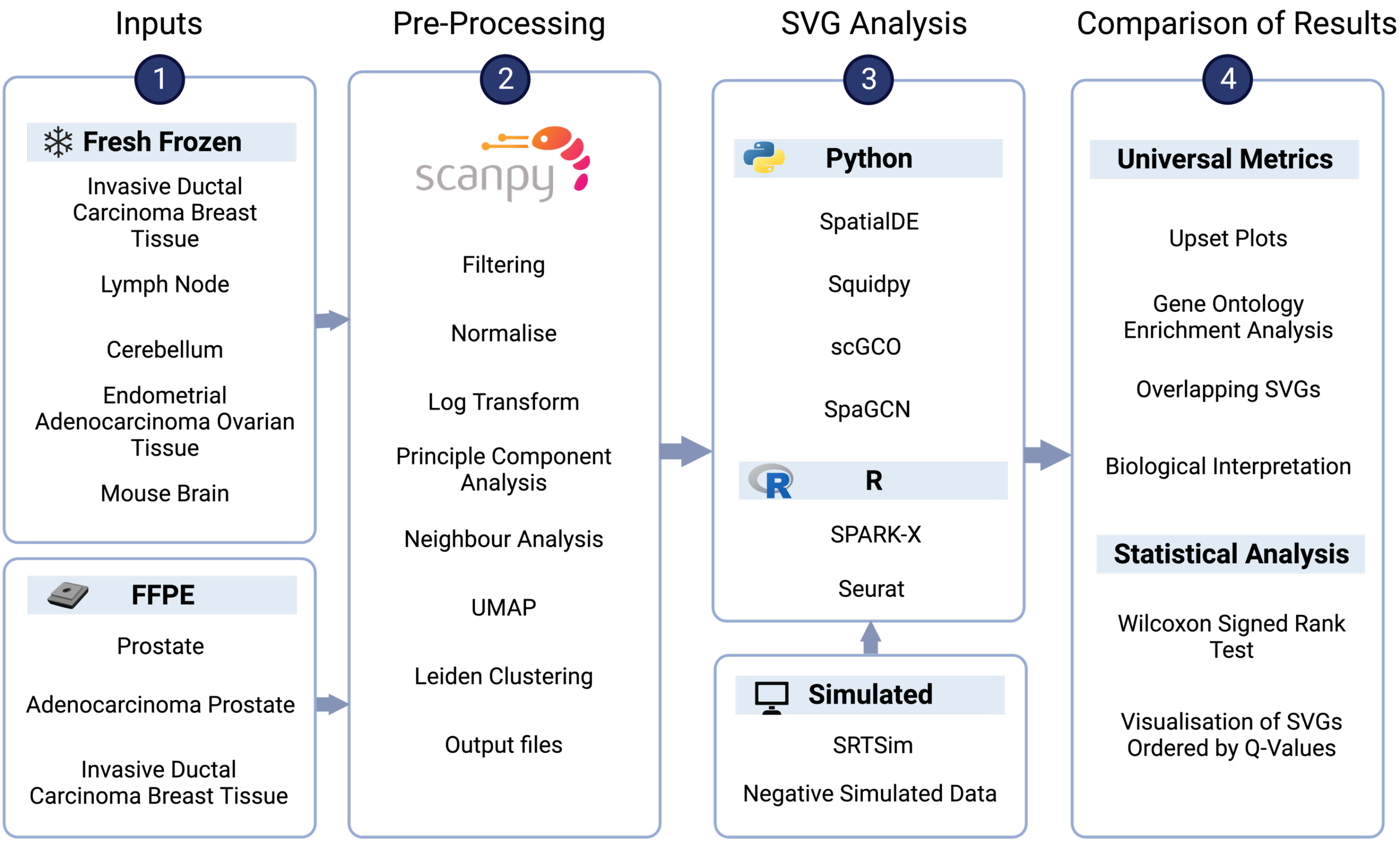

Fig S1. Overview of the design of benchmarking workflow. First, publicly available datasets are obtained to be used as inputs for pre-processing in a Scanpy workflow. For each dataset the processed output is then transformed into a data object most suitable for each of the SVG analysis packages. Simulated data is directly used as inputs for SVG analysis. Finally, the results are compared across packages within each dataset. Created with BioRender.com

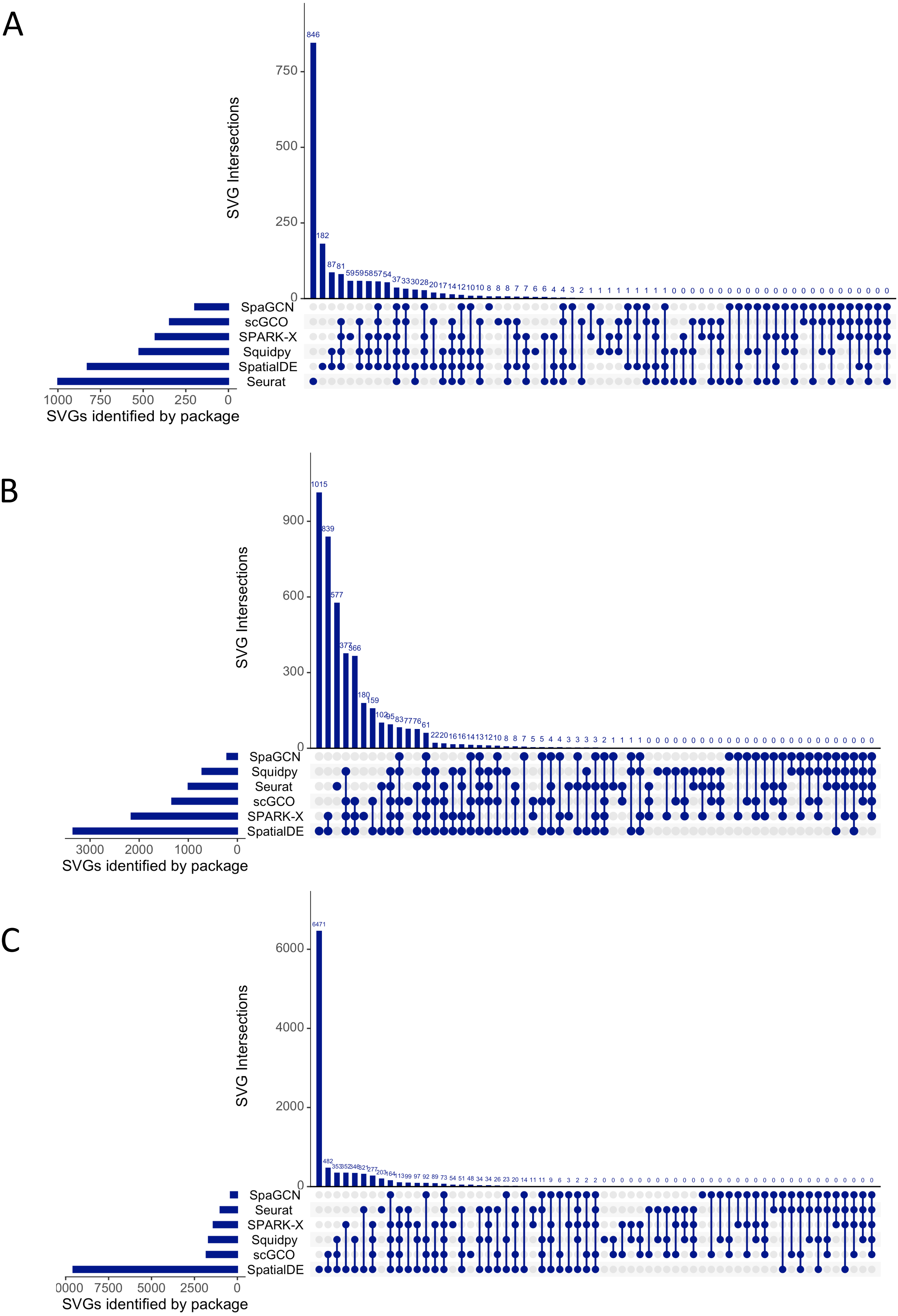
A
SVG Intersections
SpaGCN
scGCO
SPARK-X
Squidpy
SpatialDE
Seurat
SVGs identified by package
B
SVG Intersections
SpaGCN
Squidpy
Seurat
scGCO
SPARK-X
SpatialDE
SVGs identified by package
C
SVG Intersections
SpaGCN
Seurat
SPARK-X
Squidpy
scGCO
SpatialDE
SVGs identified by package

D

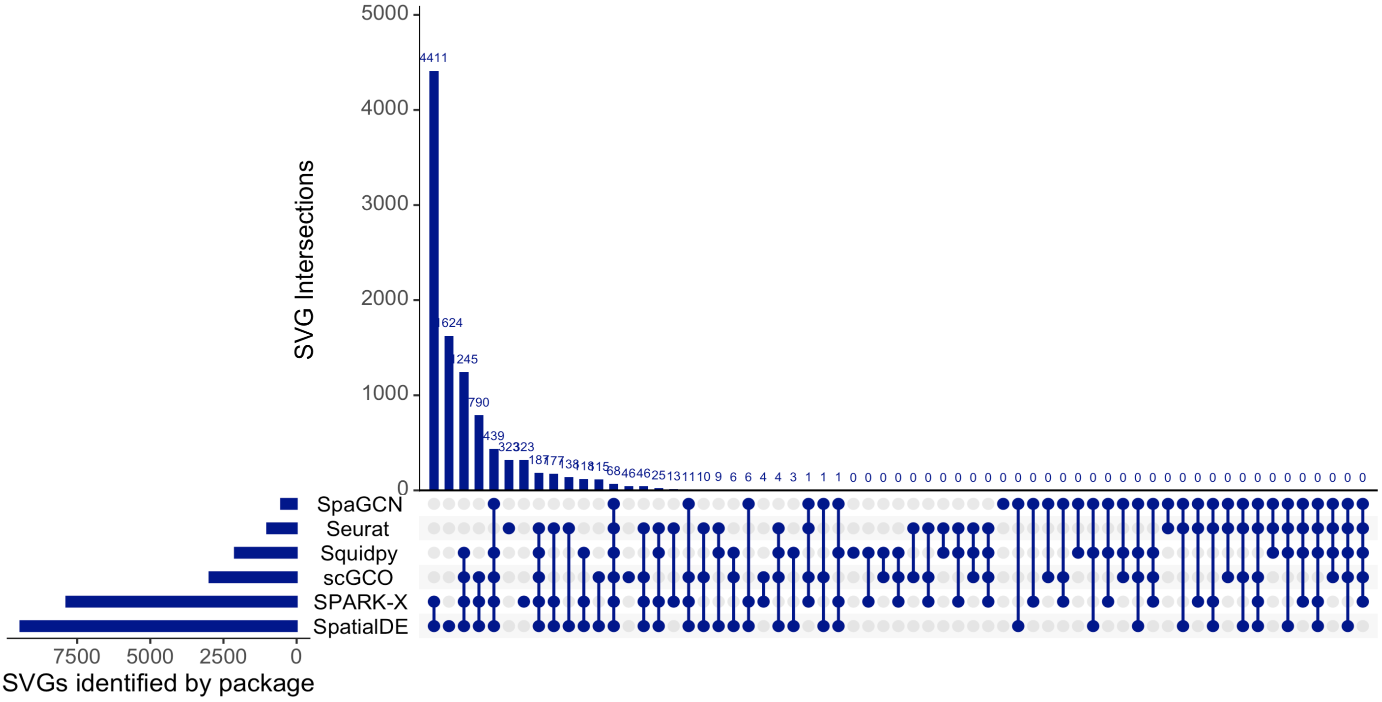

SVG Intersections
5000
4000
3000
2000
1000
0
SpaGCN
Seurat
Squidpy
scGCO
SPARK-X
SpatialDE
7500
5000
2500
0
SVGs identified by package

E

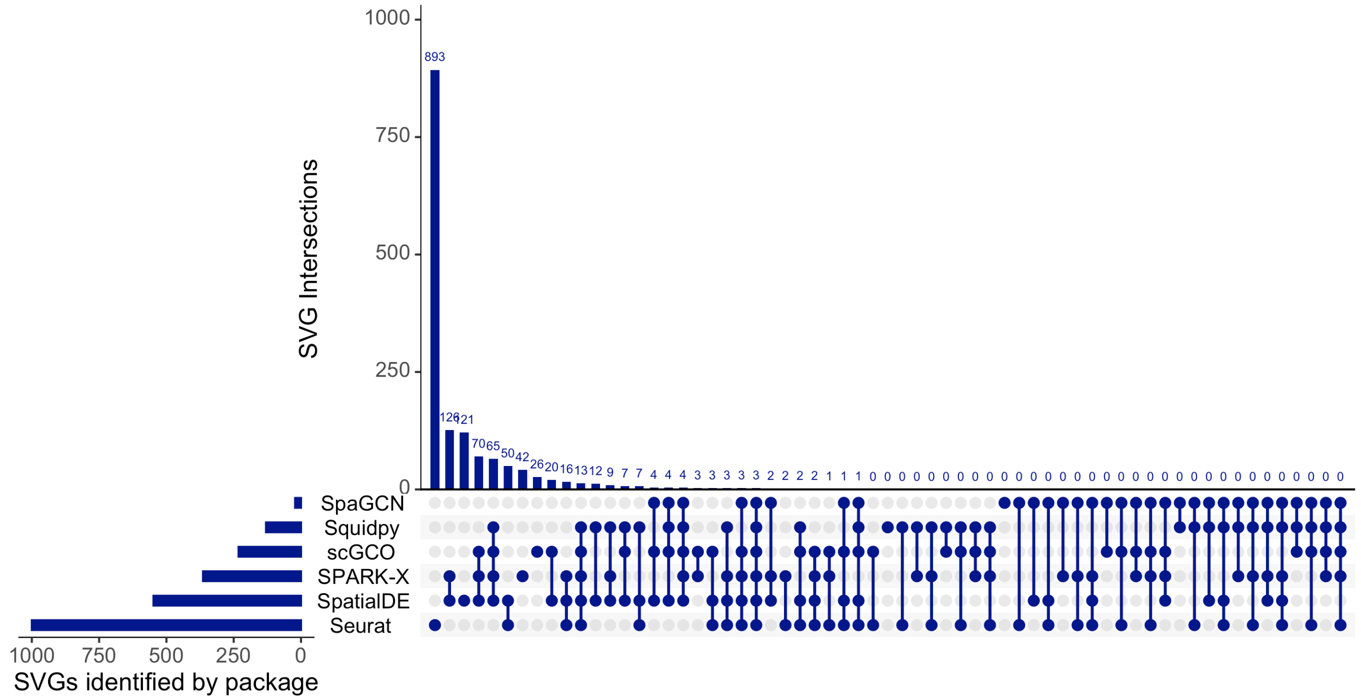

SVG Intersections
1000
750
500
250
0
893
SpaGCN
Squidpy
scGCO
SPARK-X
SpatialDE
Seurat
1000
750
500
250
0
SVGs identified by package

F

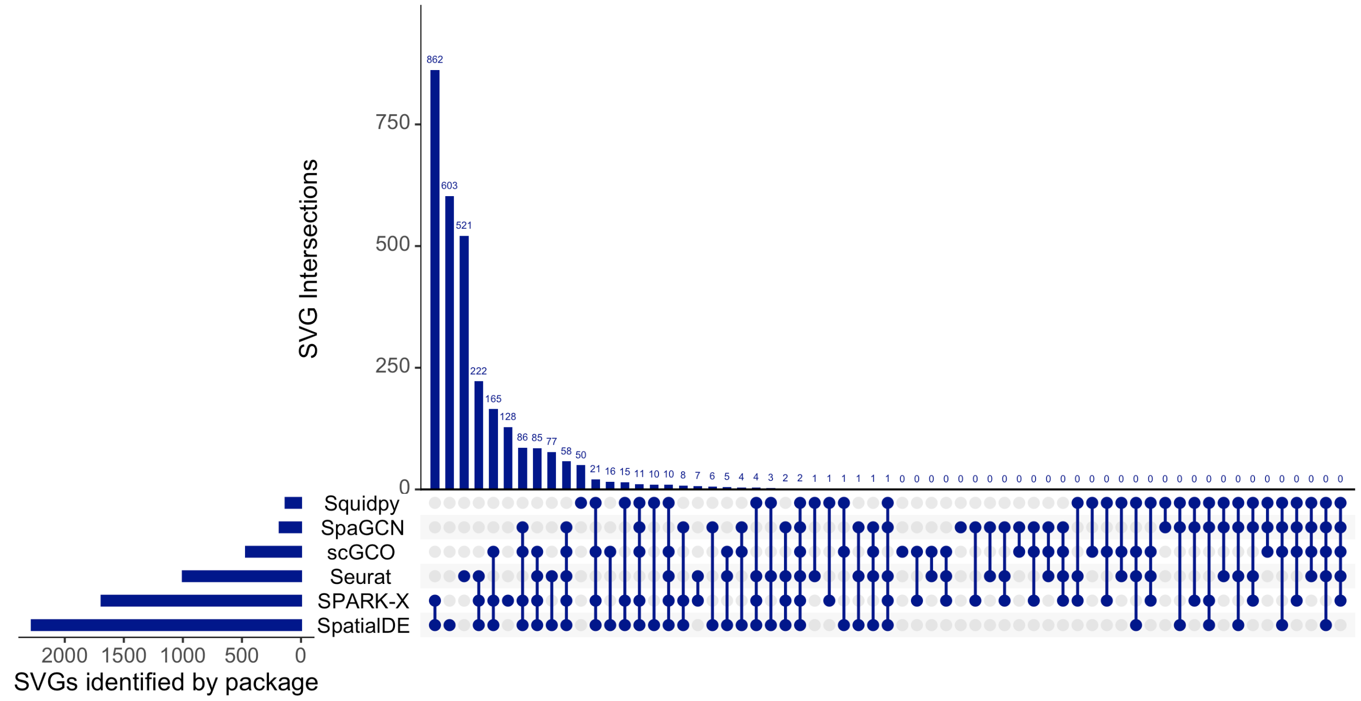

SVG Intersections
750
500
250
0
862
603
521
Squidpy
SpaGCN
scGCO
Seurat
SPARK-X
SpatialDE
2000
1500
1000
500
0
SVGs identified by package

G

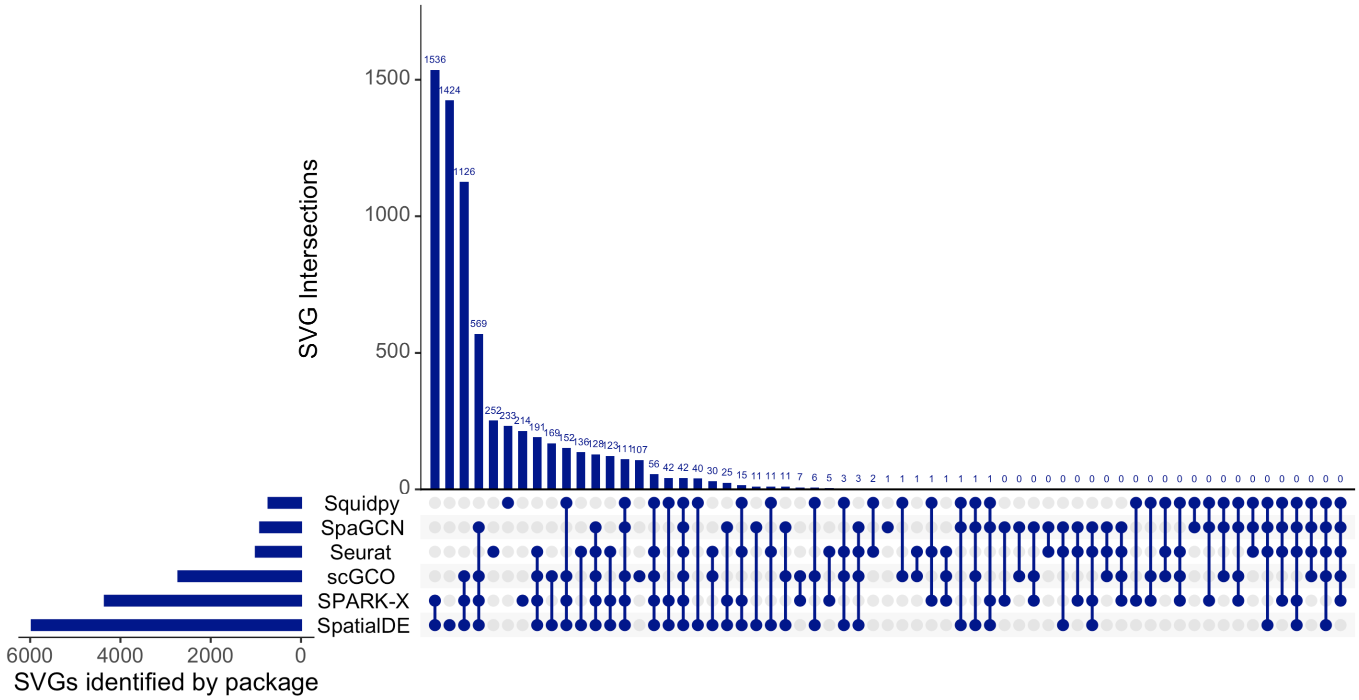

H

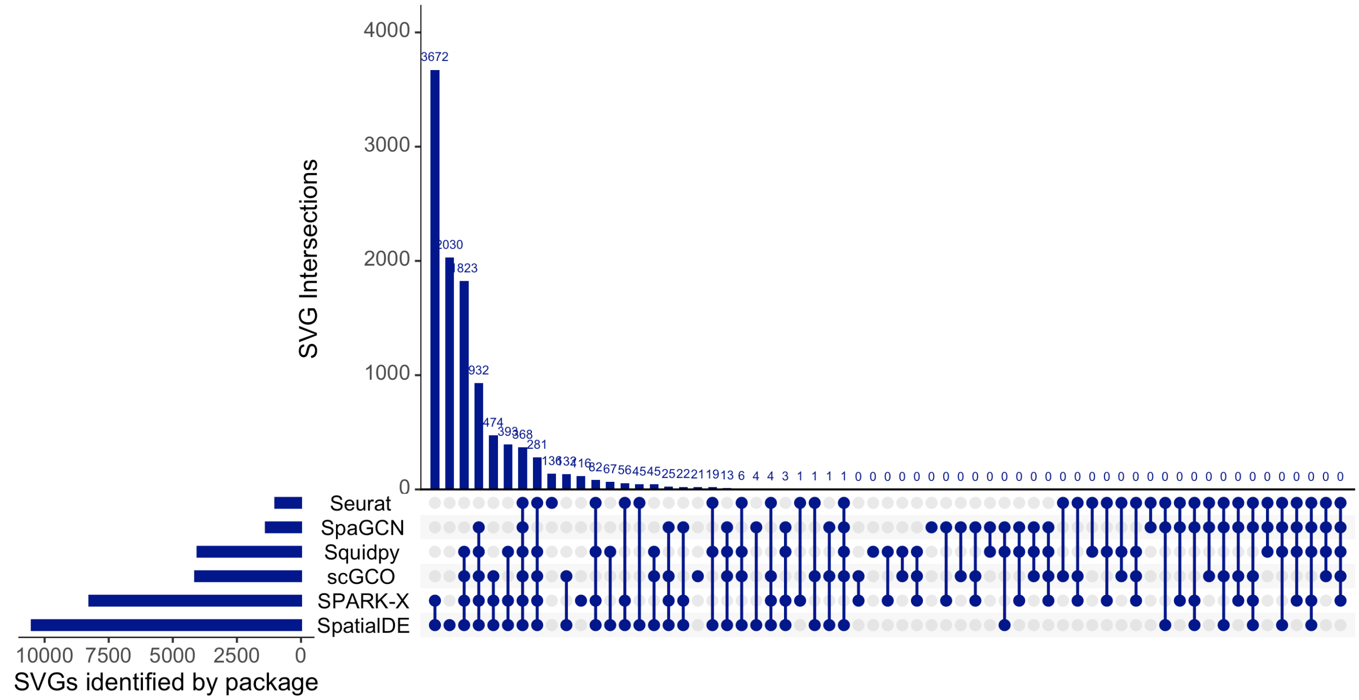

Fig S2. Distinct overlap of SVGs identified by different combinations of the six tested packages. A) FF cerebellum. B) FF lymph node. C) FFPE adenocarcinoma prostate. D) FF invasive ductal carcinoma breast tissue. E) FF left ventricle. F) FFPE prostate. G) FFPE invasive ductal carcinoma breast tissue. H) FF mouse brain coronal section.

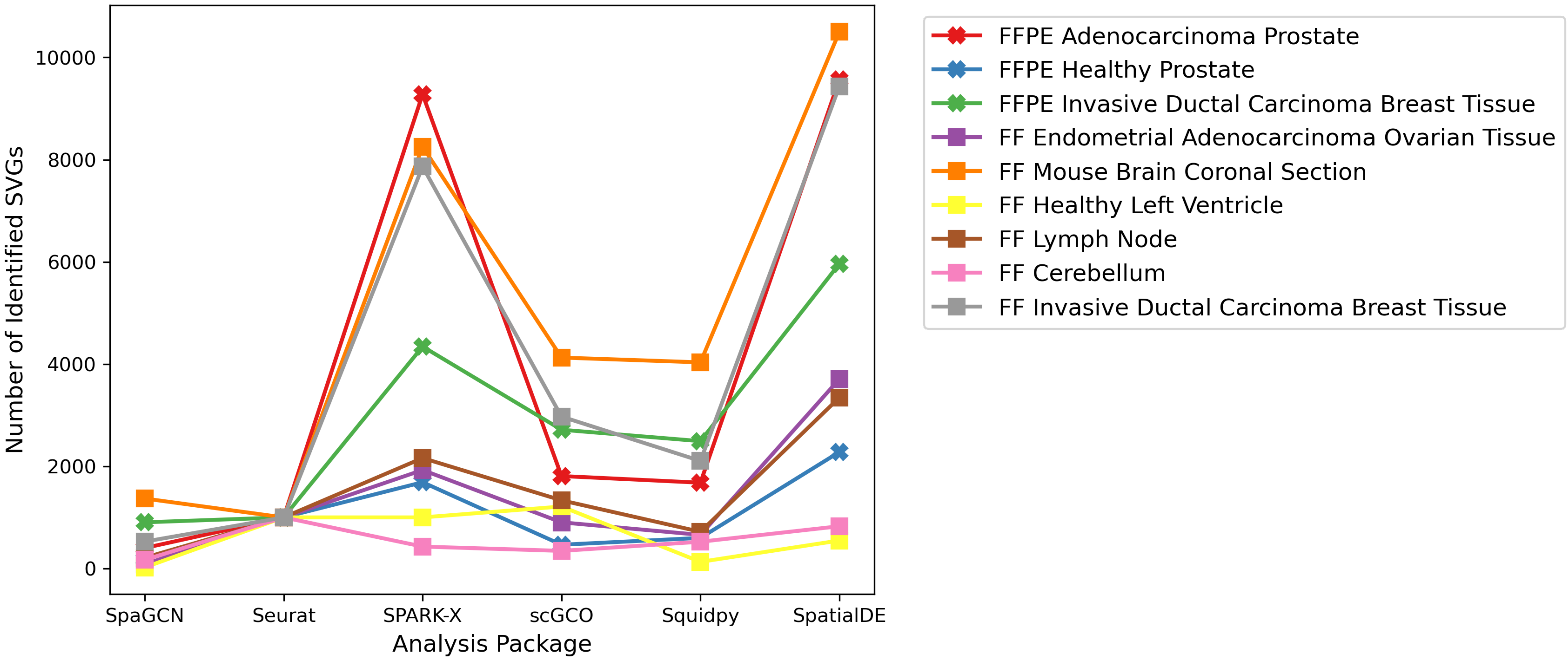

Fig S3. Comparison of the number of SVGs identified by six different packages across different datasets. FFPE tissues are visualised by a cross and FF tissues are visualised with a square. High numbers of SVGs are identified in both FF and FFPE tissues, however tissues that are more transcriptionally complex seem to have more SVGs called across the dataset. Seurat reports a consistent number of SVGs across datasets as it first identifies highly variable genes then ranks their expression by how dependent it is on spatial location (53). ç

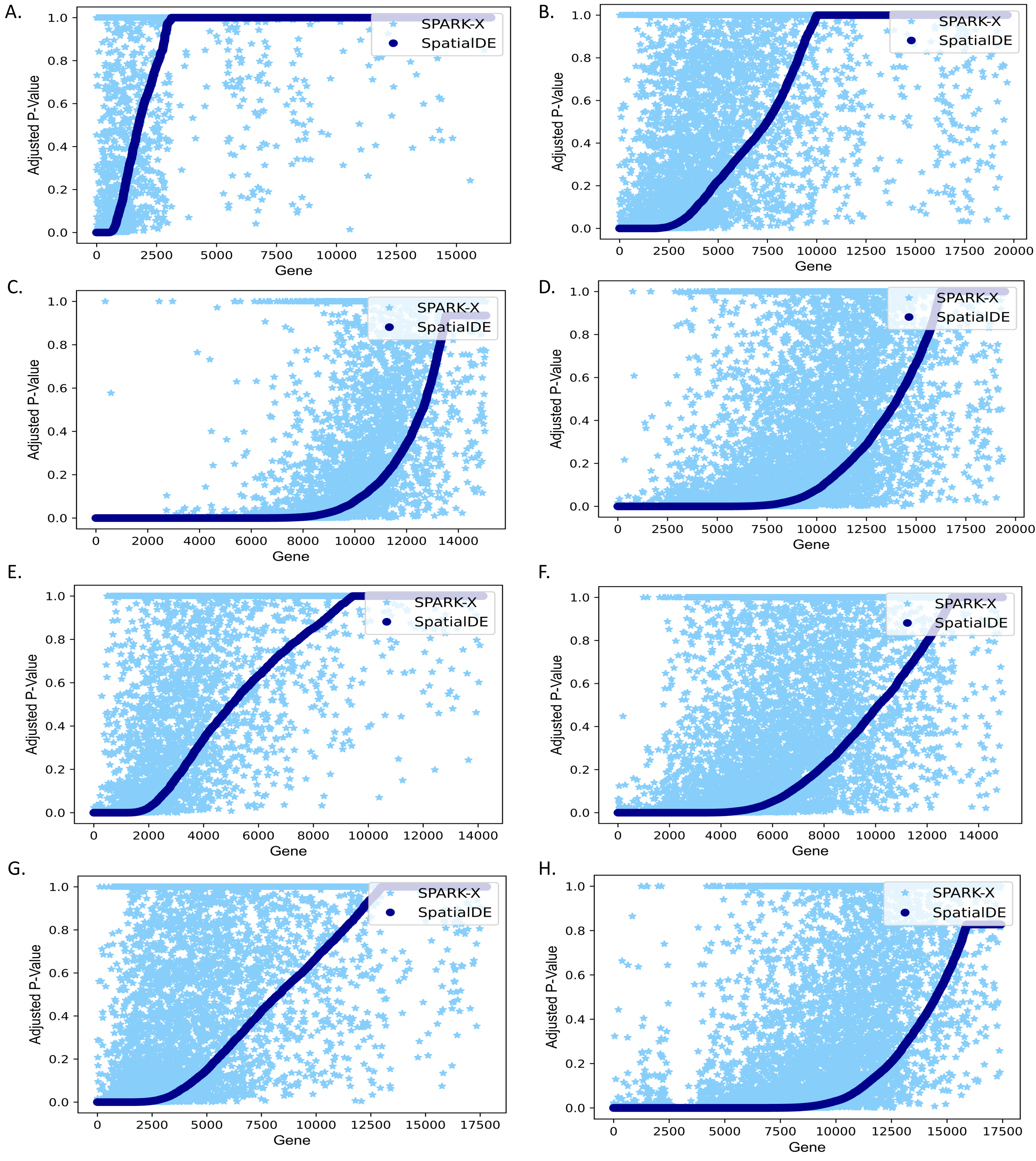

Fig S4. Comparison of ranked SpatialDE q-values against gene-matched SPARK-X q-values of all genes generated from each dataset. Order of plots repeats across all datasets. A) FF cerebellum. B) FF lymph node. C) FFPE adenocarcinoma prostate. D) FF invasive ductal carcinoma breast tissue. E) FFPE prostate. F) FFPE invasive ductal carcinoma breast tissue. G) FF endometrial adenocarcinoma ovarian tissue. H) FF mouse brain coronal section.

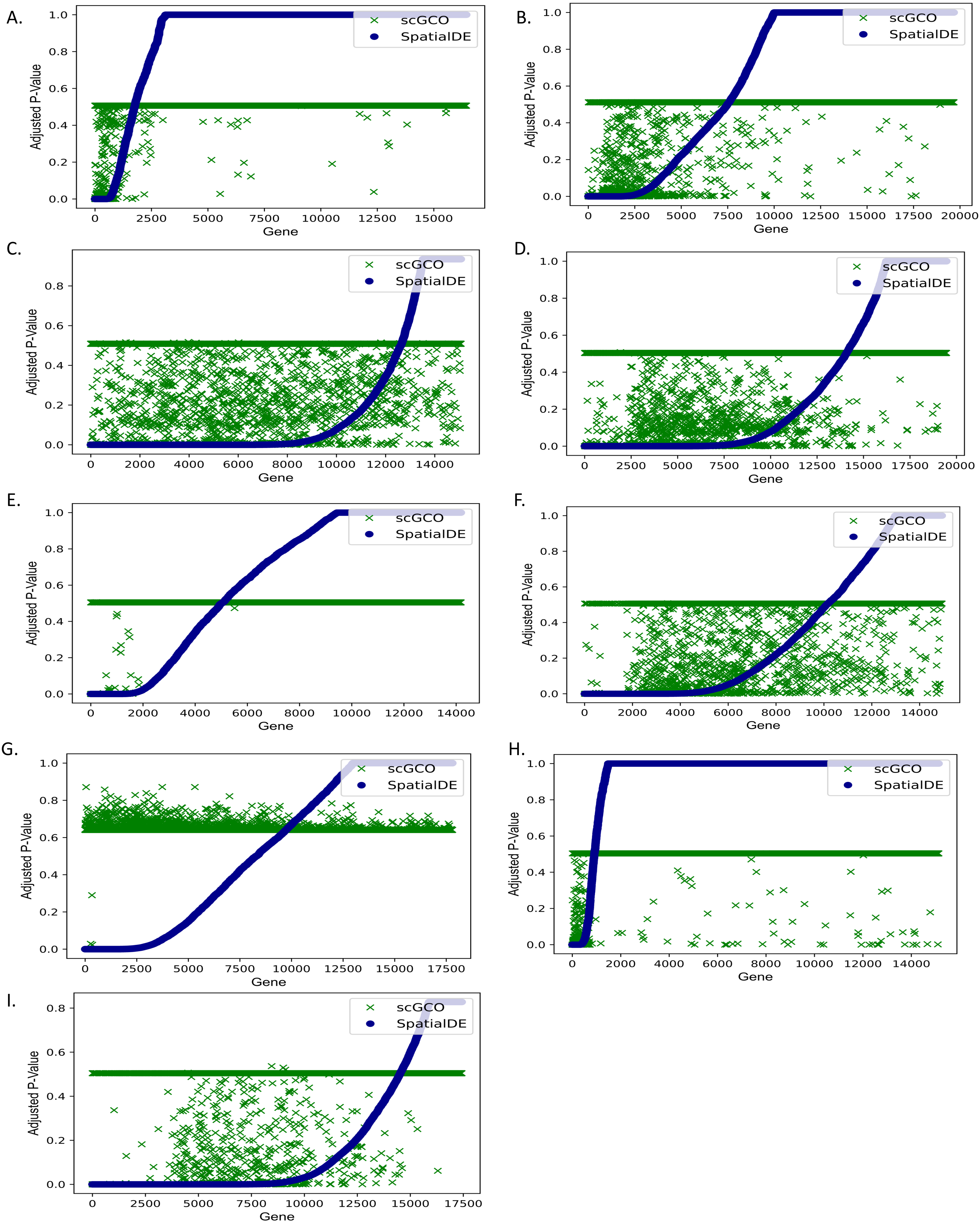

Fig S5. Comparison of ranked SpatialDE q-values against gene-matched scGCO q-values of all genes generated from each dataset. A) FF cerebellum. B) FF lymph node. C) FFPE adenocarcinoma prostate. D) FF invasive ductal carcinoma breast tissue. E) FFPE prostate. F) FFPE invasive ductal carcinoma breast tissue. G) FF endometrial adenocarcinoma ovarian tissue. H) FF left ventricle. I) FF mouse brain coronal section.

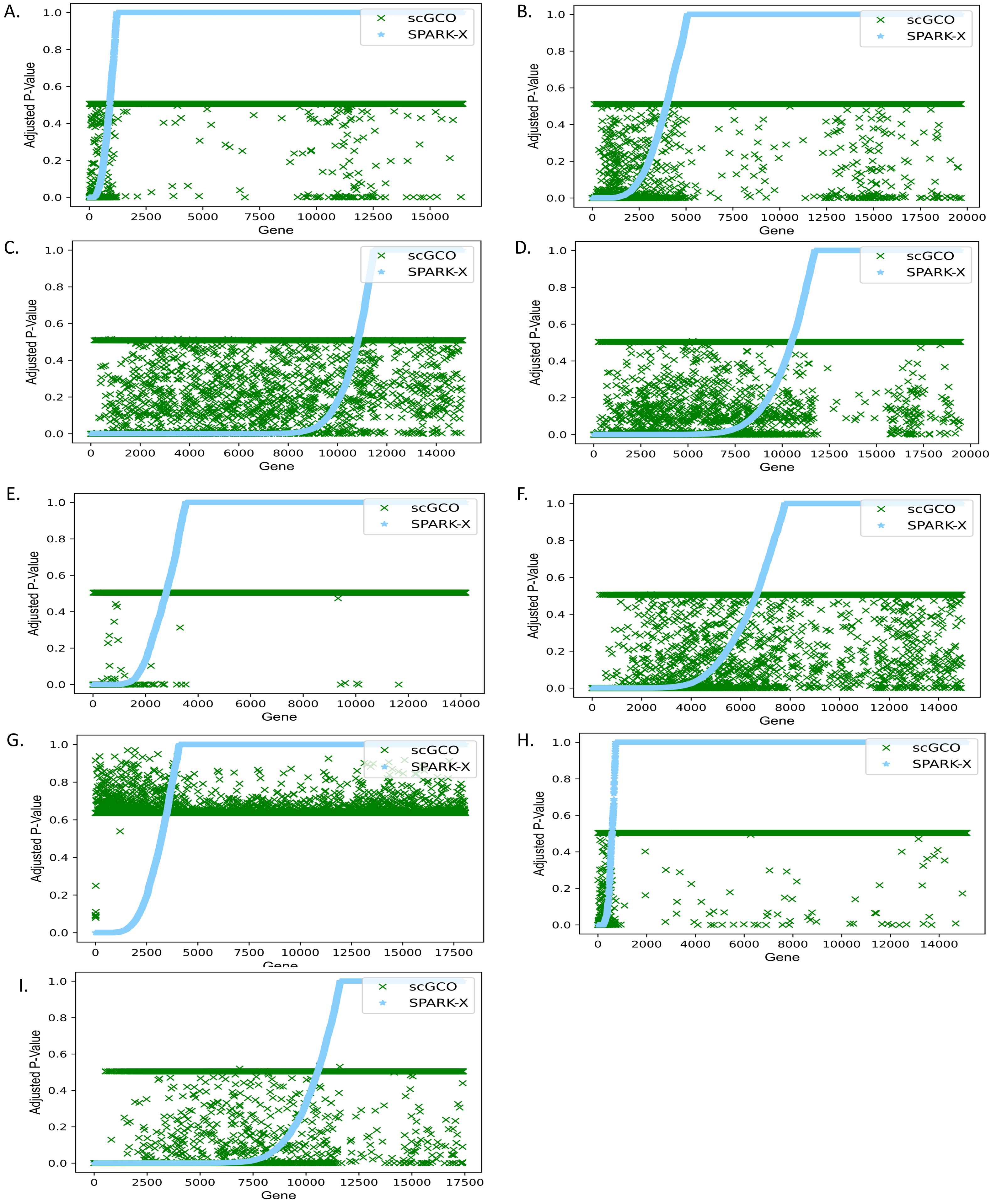

Fig S6. Comparison of ranked SPARK-X q-values against gene-matched scGCO q-values of all genes generated from each dataset. A) FF cerebellum. B) FF lymph node. C) FFPE adenocarcinoma prostate. D) FF invasive ductal carcinoma breast tissue. E) FFPE prostate. F) FFPE invasive ductal carcinoma breast tissue. G) FF endometrial adenocarcinoma ovarian tissue. H) FF left ventricle. I) FF mouse brain coronal section.

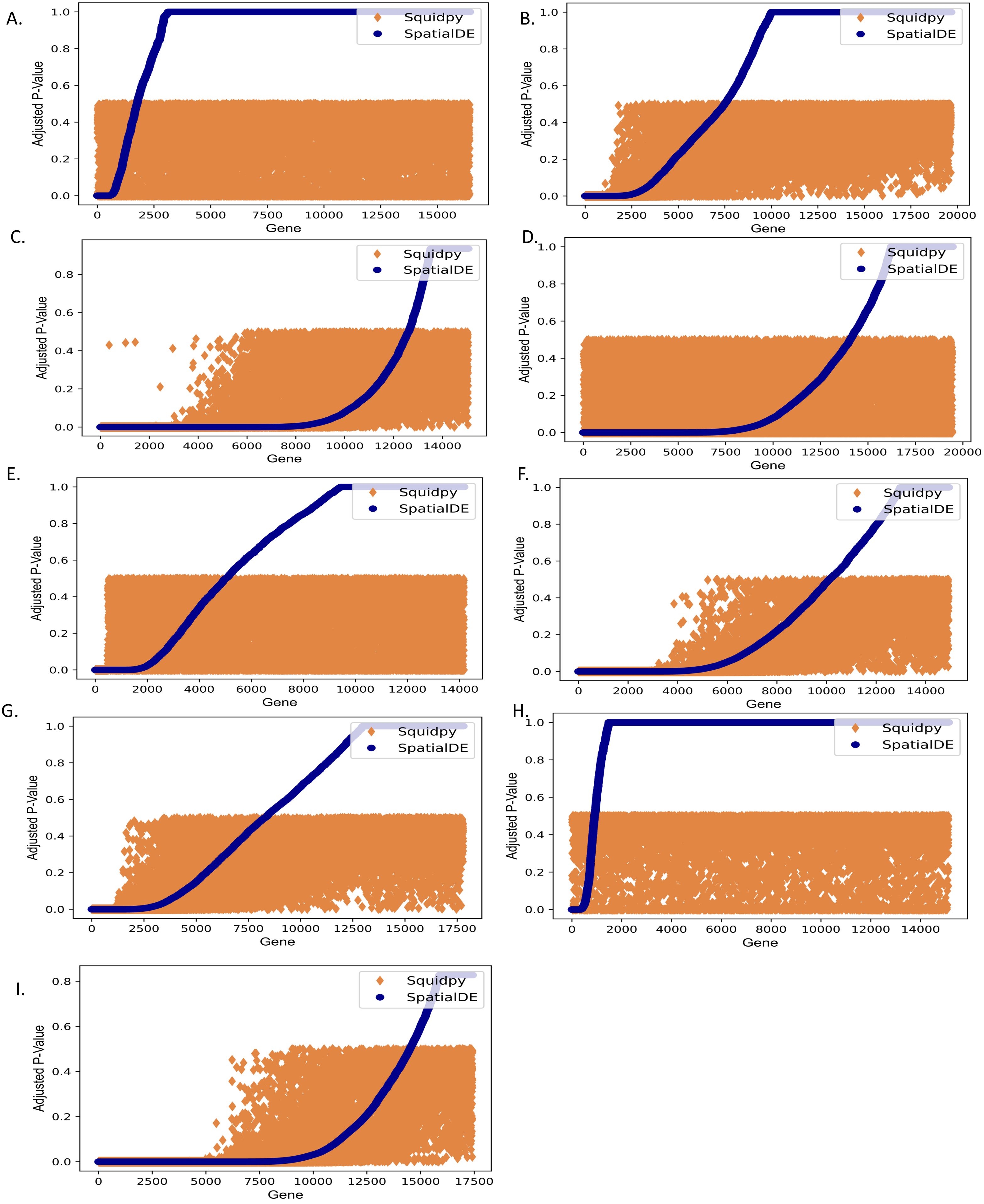

Fig S7. Comparison of ranked SpatialDE q-values against gene-matched Squidpy q-values of all genes generated from each dataset. A) FF cerebellum. B) FF lymph node. C) FFPE adenocarcinoma prostate. D) FF invasive ductal carcinoma breast tissue. E) FFPE prostate. F) FFPE invasive ductal carcinoma breast tissue. G) FF endometrial adenocarcinoma ovarian tissue. H) FF left ventricle. I) FF mouse brain coronal section.

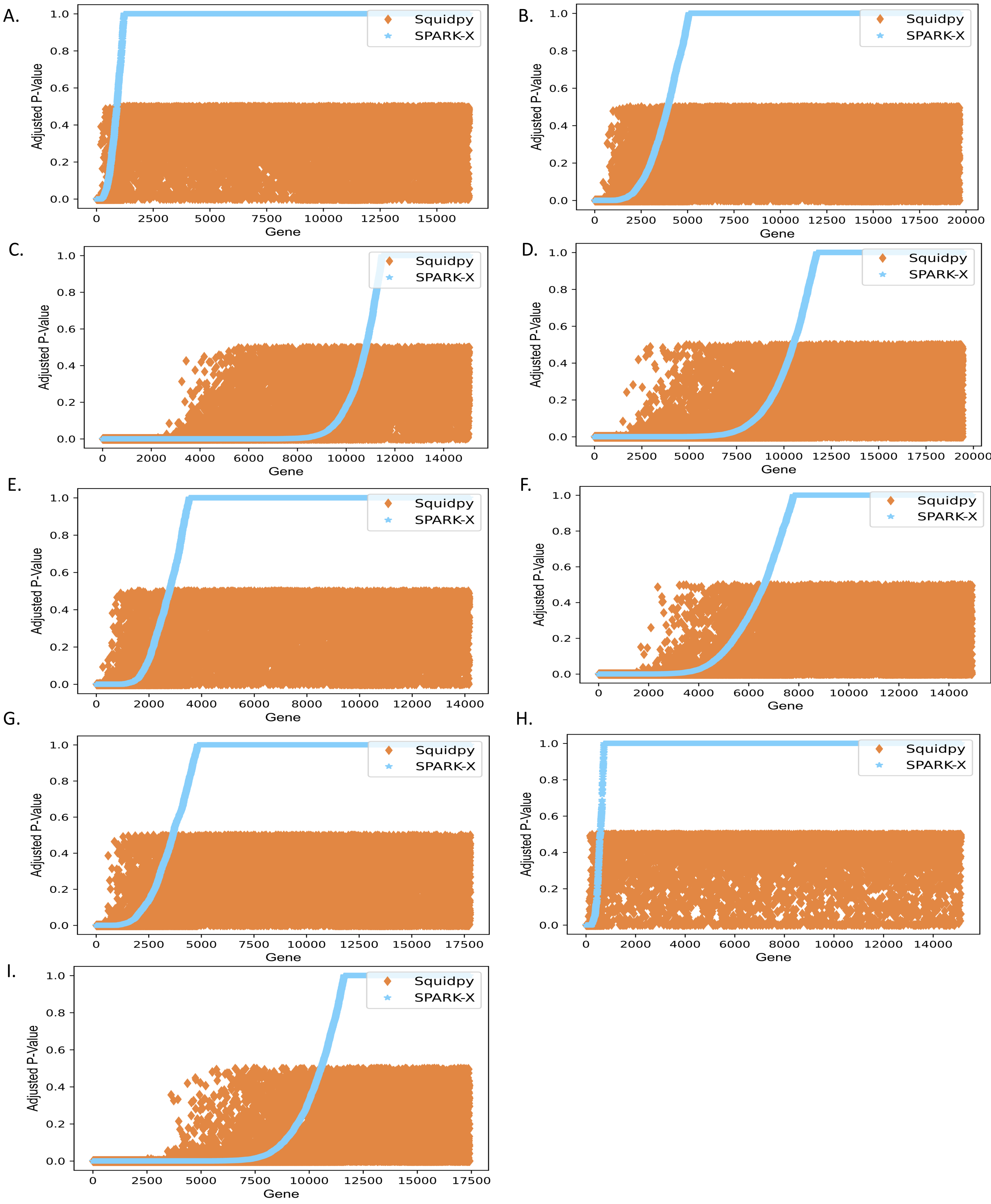

Fig S8. Comparison of ranked SPARK-X q-values against gene-matched Squidpy q-values of all genes generated from each dataset. A) FF cerebellum. B) FF lymph node. C) FFPE adenocarcinoma prostate. D) FF invasive ductal carcinoma breast tissue. E) FFPE prostate. F) FFPE invasive ductal carcinoma breast tissue. G) FF endometrial adenocarcinoma ovarian tissue. H) FF left ventricle. I) FF mouse brain coronal section.

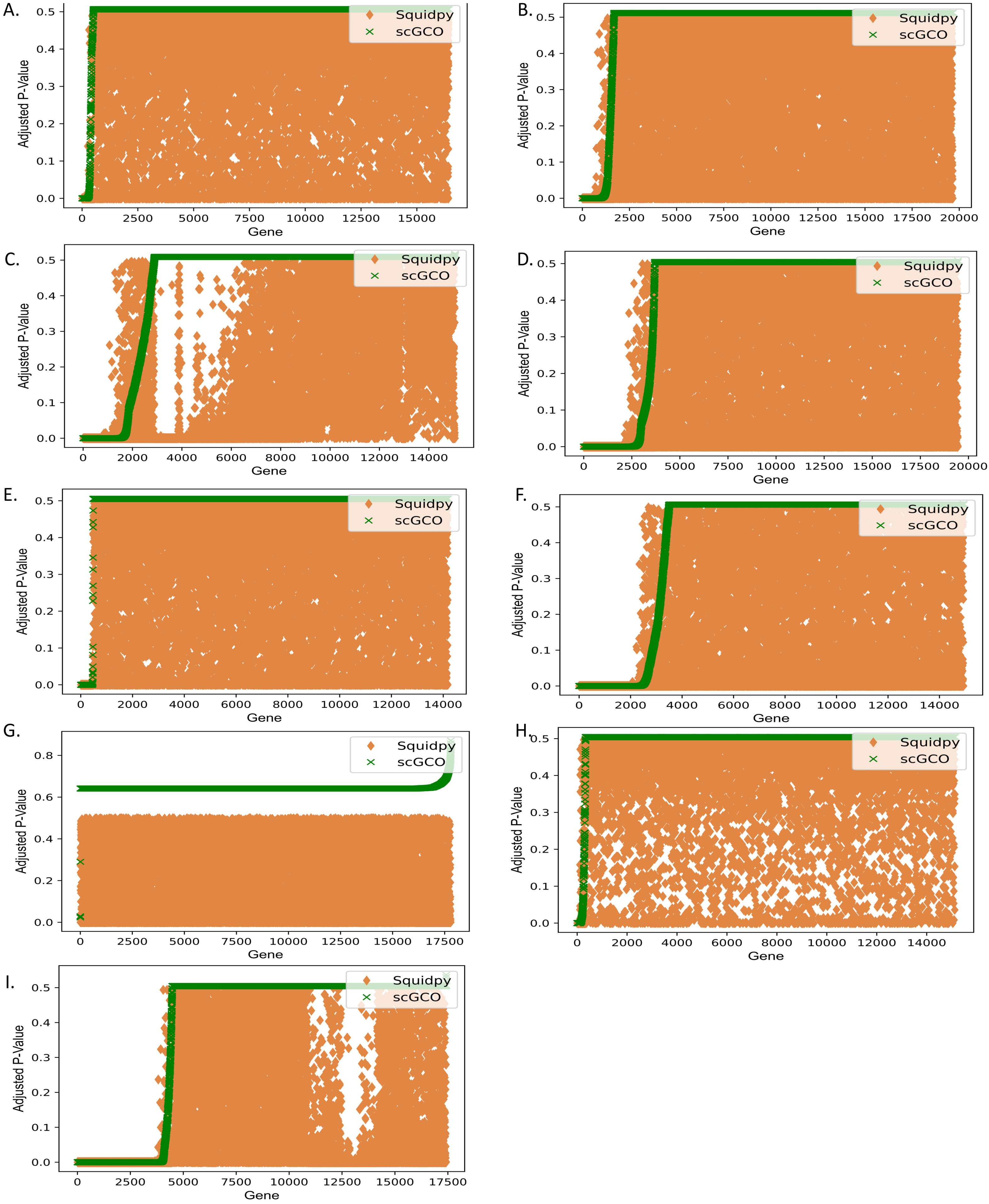

Fig S9. Comparison of ranked scGCO q-values against gene-matched Squidpy q-values of all genes generated from each dataset. A) FF cerebellum. B) FF lymph node. C) FFPE adenocarcinoma prostate. D) FF invasive ductal carcinoma breast tissue. E) FFPE prostate. F) FFPE invasive ductal carcinoma breast tissue. G) FF endometrial adenocarcinoma ovarian tissue. H) FF left ventricle. I) FF mouse brain coronal section.

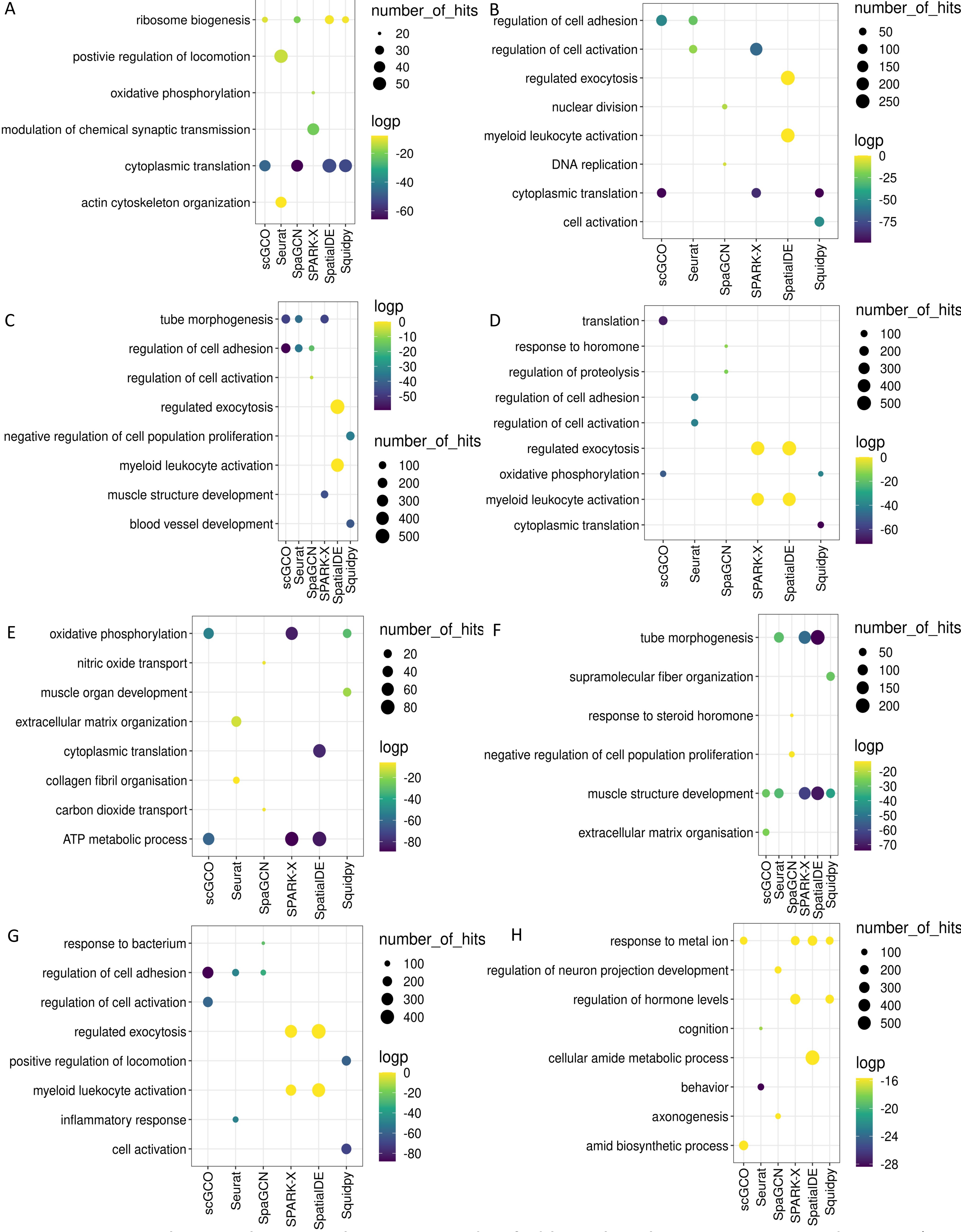

Fig S10. Gene ontology enrichment results using SVGs identified by each package as inputs across datasets. A) FF cerebellum. B) FF lymph node. C) FFPE adenocarcinoma prostate. D) FF invasive ductal carcinoma breast tissue. E) FF left ventricle. F) FFPE prostate. G) FFPE invasive ductal carcinoma breast tissue. H) FF mouse brain coronal Section.

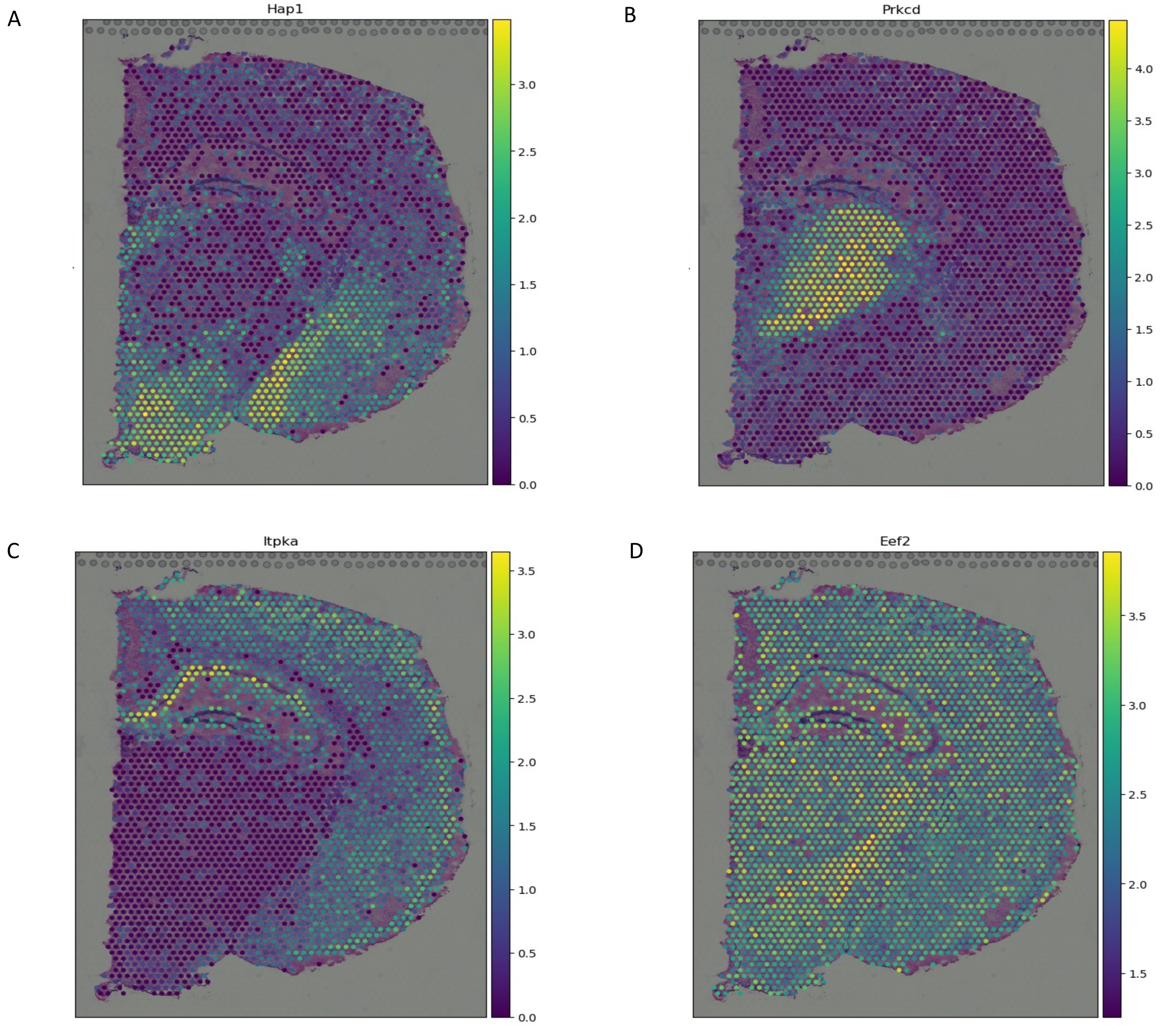

Fig S11. Spatial expression patterns of SVGs identified by all packages across the FF mouse brain coronal section dataset. A) Expression of *Hap1* across the hypothalamus and amygdala, cross-referenced with the Allen Mouse Brain Reference. B) Expression of *Prkcd* localised to the thalamus, cross-referenced with the Allen Mouse Brain Reference. C) Expression of *Itpka*, with highest expression in the isocortex, hippocampal formation (HPF) and cortical subplate consistent with patterns displayed in the Allen Mouse Brain Reference. D) Expression of *Eef2*, a known housekeeping gene in mouse (39).

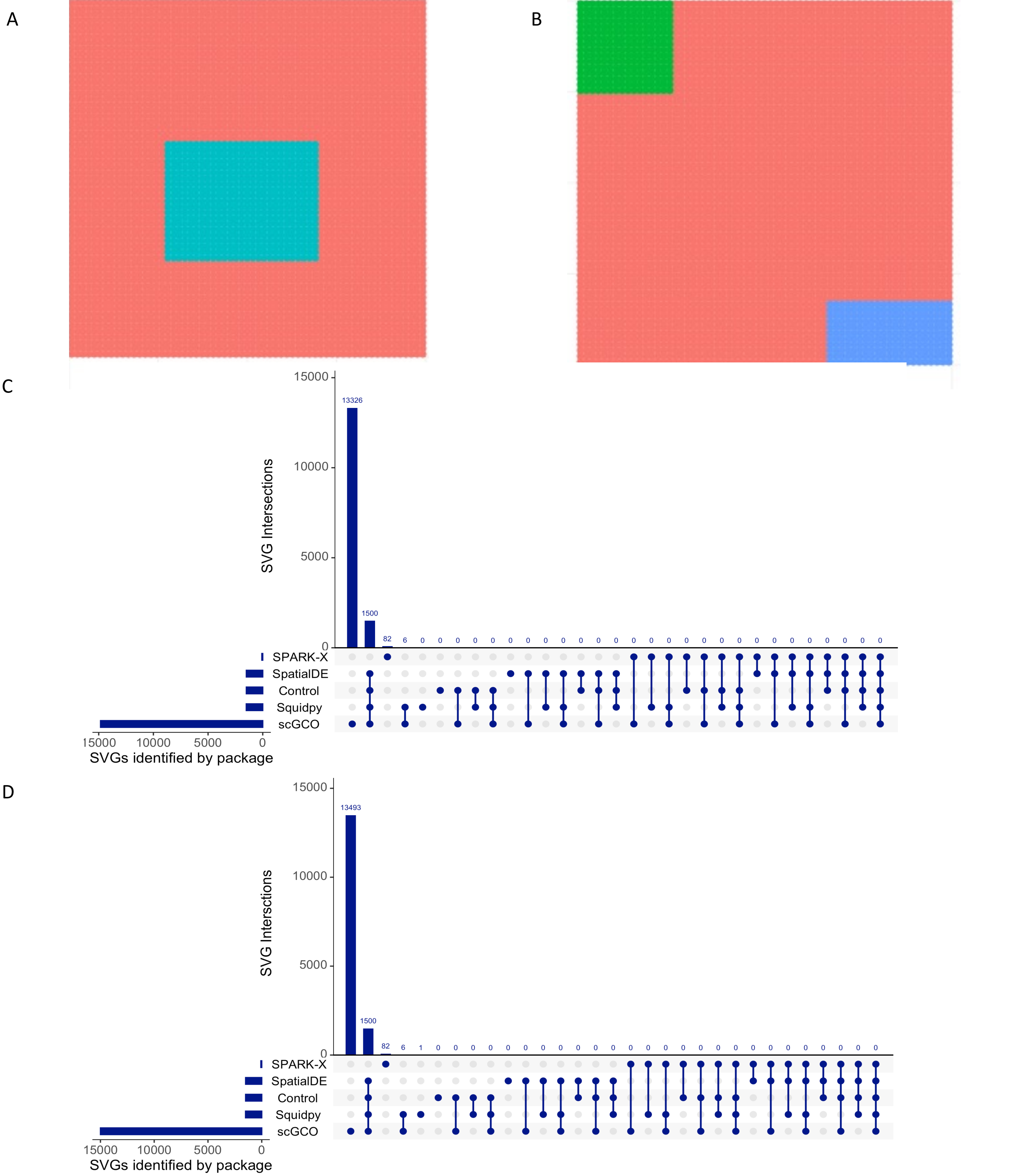

Fig S12. Simulated datasets generated with SRT sim. A) Location of simulated SVGs with a hotspot pattern visualised in blue, while red area indicates expression of noise genes. B) Location of simulated SVGs in both blue and green corners, while red area indicates expression of noise genes. C) Distinct overlap of SVGs compared to the control SVG list identified by different combinations of the four tested packages. 1500 SVGs were present in this dataset. D) Distinct overlap of SVGs compared to the control SVG list identified by different combinations of the four tested packages. 750 high signal SVGs and 750 low signal SVGs were present in this dataset.

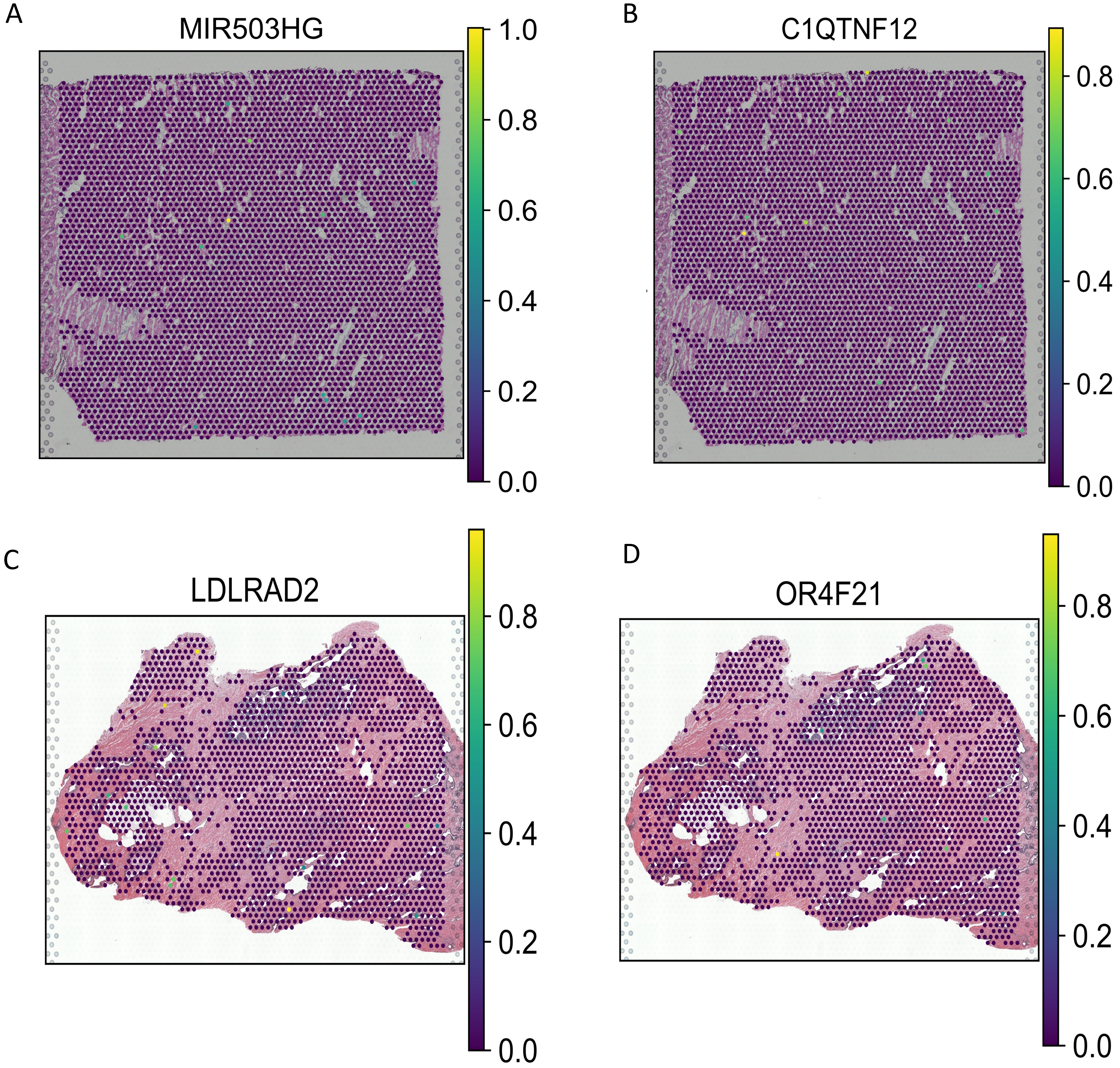

Fig S13. Top SVG identified by SpatialDE in negative simulated datasets generated by A) Randomising coordinates of FF Left Ventricle dataset. B) Randomising counts and coordinates of FF left ventricle dataset. C) Randomising coordinates of FFPE prostate dataset. D) Randomising counts and coordinates of FFPE prostate dataset.

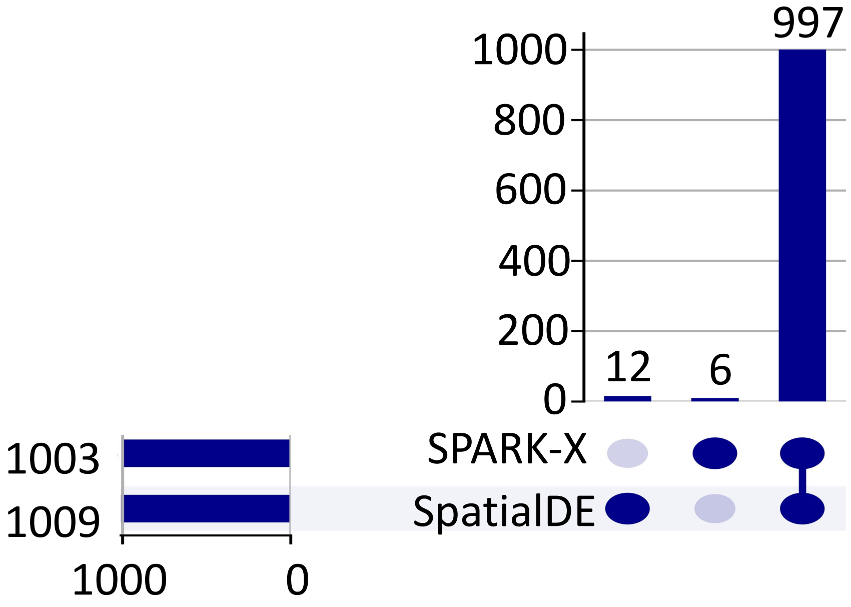

Fig S14. Upset plot of results generated from running SPARK-X and SpatialDE on simulated data with known pattern of SVGs.
