# Supplementary material for: Disparities in spatially variable gene calling highlight the need for benchmarking spatial transcriptomics methods
Source: Genome Biol. 2023 Sep 18;24:209. doi: 10.1186/s13059-023-03045-1 (PMC10506280; doi:10.1186/s13059-023-03045-1)
Supplement: Supplementary file 3 — Additional file 3. Review history [file 13059_2023_3045_MOESM3_ESM.docx]

Review History

**First round of review**

**Reviewer 1**

**Were you able to assess all statistics in the manuscript, including the appropriateness of statistical tests used?**

Yes, and I have assessed the statistics in my report.

**Comments to author:**

In this manuscript the authors reported results to suggest an important message about the extent of inconsistency in different methods to find most spatially variable genes (SVG) across a tissue section in a spatial dataset. Finding SVG is one of the common analysis tasks for a spatial dataset, so it is important for the community to appreciate the variation in methods and know about good analysis practice to find SVG.

Main questions and suggestions:

- I suggest adding a diagram for a benchmarking experiment design to include methods, datasets, assessment metrics. More discussion about assessment metrics would be helpful.

- I suggest adding text or a figure panel summarising the similarity/differences/grouping of the statistical algorithms used in the five methods.

- For this work, it is difficult to establish a ground truth to assess sensitivity and specificity. The authors used simulated datasets and discussed several caveats of such simulation. The authors may consider investigating samples where the genes are known to express differently from one anatomical region to another. It would also be useful to consider genes that are known to be stable across regions (e.g. would housekeeping genes like ACTB stay constant spatially?)

- Among the 8 datasets tested, how does the performance change relative to the complexity of the tissue sections? One would expect that more complex tissue, like cancer would have more SVG? I suggest discussing more about this in relation to the datasets tested.

- It would be helpful to provide some information on best practice, for example a decision tree on in which case and/or for which datasets, users should use which method.

Minor comments:

- "The goal of SRT analysis pipelines is the robust and reliable identification of spatially variable gee (SVGs) within tissue sections (12,13)". I think there are many other goals. Please revise this sentence.

- "We observed non overlapping parent terms between datasets in the top 10 over-represented GO terms (Fig. 1D)"-> should this be Fig 1B?

- Figure 1D show an example of a HVG gene. It is good to also include an example of a gene that is not spatially variable.

**Reviewer 2**

**Were you able to assess all statistics in the manuscript, including the appropriateness of statistical tests used?**

Yes, and I have assessed the statistics in my report.

**Comments to author:**

The manuscript by Charitakis et al. describes a benchmarking analysis across five different software for analysis of spatially resolved transcriptomics datasets: SpatialDE, SPARK-X, Seurat, SpaGCN and scGCO. Specifically, the authors investigate the overlap across spatially variable genes (SVGs) identified by the five methods and find that they do not provide a consistent overlap among the five of them if not for genes with a stronger signal to noise ratio. In addition, the authors validate their results by using two negative control datasets obtained by permutation of spatial coordinates and gene expression matrix. From this analysis,	Seurat, scGCO and SPARK-X identified no SVGs, indicating an ability to distinguish signal from noise, which is highly encouraging.

Finally, the authors leverage two types of datasets: fresh frozen (FF) and formalin-fixed paraffin embedded (FFPE) tissues.

This work is timely. With the increasing amount of spatial datasets available, it is highly valuable to identify appropriate tools for spatial analyses. However, I do have some major concerns:

- SpatialDE is a relative old software (2018) which is not maintained to my knowledge. It is also the software that gives the most discrepancies. I recommend the authors to benchmark more recent software like Squidpy.

- While the authors reach the point of performing GO analysis of the SVGs to investigate ontology overlap across software, more thorough analysis should be carried out by investigating the spatial distribution of the SVGs deriving from the different software.

- It would be helpful for general readers to have a brief summary of the differences in identifying SVGs across the five benchmarked methods.

- Some extra considerations between FF and FFPE material to calculate SVGs would be appropriate.

Minor concern:

- line 25: FF should stand for fresh frozen instead of formal fixed.

**Authors Response**

**Point-by-point responses to the reviewers’ comments:**

**Response to Reviewers:**

We thank the Reviewers for their considered and constructive reviews. We note that both Reviewers recognise the need for robust benchmarking for spatial transcriptomic profiling and appreciate their positive response to the manuscript. To address the concerns raised by the Reviewers, we have included new analysis tools, provided additional new data, and modified the manuscript. The revised manuscript is considerably improved as a result. The key additions to the manuscript are:

- we have further uncovered the basis of the disparities between methods for calling spatially variable genes, achieved through the generation of two new simulated datasets applied on four different packages to calculate specificity and sensitivity,
- we have strengthened our analysis by adding one more additional package (Squidpy) to the original five tested and one additional public, well-annotated dataset (fresh frozen mouse brain coronal section),
- we have further expanded our benchmarking of the software packages used through new comparison criteria.

Specific responses to the Reviewers’ concerns are given below.

**Reviewer #1:**

**In this manuscript the authors reported results to suggest an important message about the extent of inconsistency in different methods to find most spatially variable genes (SVG) across a tissue section in a spatial dataset. Finding SVG is one of the common analysis tasks for a spatial dataset, so it is important for the community to appreciate the variation in methods and know about good analysis practice to find SVG.**

**Main questions and suggestions:**

**Comment 1: I suggest adding a diagram for a benchmarking experiment design to include methods, datasets, assessment metrics. More discussion about assessment metrics would be helpful.**

**Response 1:** We thank the reviewer for their comments, we agree that providing a schematic would provide a useful resource. We have included a new figure outlining the experimental design of the benchmarking method as suggested. This figure is included in the manuscript as Additional File 1: Fig. S1 and reproduced below.

Fig S1. Overview of the design of benchmarking workflow. First, publicly available datasets are obtained to be used as inputs for pre-processing in a Scanpy workflow. For each dataset the output is then transformed into a data object most suitable for each of the SVG analysis packages. Simulated data is directly used as inputs for SVG analysis. Finally, the results are compared across packages within each dataset.

**Comment 2: I suggest adding text or a figure panel summarising the similarity/differences/grouping of the statistical algorithms used in the five methods.**

**Response 2:** The six packages chosen for this study were selected to demonstrate how different mathematical models are suited to labelling SVGs within Visium datasets. In line with the reviewer’s suggestion, we have added a section discussing the differences in more detail in the Supplementary Methods, lines 10-43 (Additional File 18). The section is also included below:

“Six state of the art packages built for the identification of SVGs (Seurat and Squidpy excepted) in SRT data were selected for benchmarking: SpatialDE, SPARK-X, Seurat, SpaGCN, Squidpy and scGCO. Each package uses a different algorithm to identify SVGs and holds varied assumptions on the distribution of gene expression data (Additional File 12: Table S3). Packages were purposefully chosen to compass a variety of algorithms and associated mathematical models of gene distribution expression that can be used to identify these spatial patterns. While two packages used algorithms based on graph theory, the others were selected to test different mathematical assumptions regarding SRT data. SpatialDE employs Gaussian process regression, a non-parametric probabilistic model (Additional File 2: Table S3) (1). SPARK-X is another non-parametric method, building on a covariance test framework, specifically the projection covariance function (2). This function can measure similarity between two locations or gene expression, it can quantify if the product of the two inputs deviates significantly from the mean value (2). Seurat employs a mark point process, first used by the Trendsceek package (3,4). This is another non-parametric approach that can test if gene expression levels are significantly dependent on the spatial distribution of spots as a function of the distance between them (3). This can then calculate the mark-variogram (3,4). SpaGCN’s method is built around a graph convolutional network (GCN)-based approach and is unique in that it incorporates signal from histology images and restricts identification of SVGs to within spatial domains (5). Gene expression and image data are converted into a weighted undirected graph, which then calculates the distances between any two vertices in the graph (5). The nodes represent spots, and the edge weight is calculated using the histology image and the Euclidian distance between two vertices (5). The weight of each edge is calculated as a function between how related spots are in the graph (5). Dimensionality reduction then graph convolution is computed to then identify spatial domains (5). Finally, differential expression analysis is performed between spots in one spatial domain with neighbouring domains utilising a Wilcoxon rank-sum test and genes with an adjust p-value < 0.05 are reported as SVGs (5). scGCO is another graph-based method, but this package utilises a probabilistic graph model by optimising hidden Markov random fields (HMRF) for the purpose of SVG identification (6). Similar to Seurat, scGCO uses a marked point process to model spatial gene expression (4,6). The dependency of gene expression states on spatial locations is analysed using the complete spatial randomness framework, and scGCO overcomes its limitations using HRMF (6). Finally, Squidpy employs the spatial autocorrelation metrics of Moran’s I to label SVGs (7). Given a continuous feature, in this case gene expression level and their spatial location it can evaluate whether a pattern is present or not using Moran’s I (7).”

A table summarising some of the differences has also been included (Additional File 12: Table S3) and is featured below. Rows highlighted in blue indicate packages using algorithms based on graph theory.

Table S3) Overview of publicly available 10X Visium datasets to be included in benchmarking process. FF indicates tissues that are fresh frozen and FFPE indicates tissues that are formalin-fixed paraffin-embedded. The filtered output files and imaging data from the Space Ranger v1.0.0, v1.2.0 or V1.3.0 pipeline were downloaded for each dataset.

**Comment 3: For this work, it is difficult to establish a ground truth to assess sensitivity and specificity. The authors used simulated datasets and discussed several caveats of such simulation. The authors may consider investigating samples where the genes are known to express differently from one anatomical region to another. It would also be useful to consider genes that are known to be stable across regions (e.g. would housekeeping genes like ACTB stay constant spatially?)**

**Response 3:** We thank the reviewer for this great suggestion. We have taken two orthogonal approaches to address sensitivity and specificity of each package, first by analysing samples with known regions of specific gene expression as a positive control and samples with no changes in expression as negative controls. The samples included to address the suggestion are as follows:

Positive controls:

1. A sample of 10X Visium Fresh Frozen Coronal Section of the adult mouse brain.
2. Simulated datasets with known SVGs were generated using the SRTsim package.

Negative control:

1. Simulated datasets with known genes that do not display a spatial pattern were generated using the SRTsim package.
2. Publicly available datasets were permutated to remove any spatial gene expression signal, included in the previous version of the manuscript.

The results from the analysis of the FF mouse brain coronal section, a tissue with spatially distinct and well-annotated regions, is included in the revised manuscript lines 70-74 and Additional file 1: Fig S11. The updated text and figure are also included below.

“Furthermore, in FF Mouse Brain Coronal Section, a dataset with known spatial gene expression patterns, many of the 368 commonly identified SVGs display expression patterns corresponding to their known region of expression in the Allen Mouse Brain Atlas (Additional File 1: Fig S11 A-C) (37). These observations indicate that patterns with a stronger signal-to-noise ratio are identified by all methods.”

Fig S11. Spatial expression patterns of SVGs identified by all packages across the FF mouse brain coronal section dataset. A) Expression of *Hap1* across the hypothalamus and amygdala, cross-referenced with the Allen Mouse Brain Reference. B) Expression of *Prkcd* localised to the thalamus, cross-referenced with the Allen Mouse Brain Reference .C) Expression of *Itpka*, with highest expression in the isocortex, hippocampal formation (HPF) and cortical subplate consistent with patterns displayed in the Allen Mouse Brain Reference. D) Expression of *Eef2*, a known housekeeping gene(37).

Notably, many of the overlapping SVGs from all six packages, such as *Hap1, Prkcd* and *Itpka* display clear spatial patterns. However, the introduction of false positives by four of the packages is noted with the annotation of the housekeeping gene *Eef2* as an SVG (Additional File 1: Fig S11D) Further details are in lines 104-110 of the revised manuscript. The corresponding lines from the manuscript have been included below and panel refers to panel D in the figure above.

“Inconsistencies in the results between different packages may therefore be explained by the differences in FP rates, rather than the rate of false negatives. To investigate this, expression of known housekeeping gene *Eef2* (39) was visualised across the FF Mouse Brain Coronal Section dataset to verify its ubiquitous expression (Additional File 1: Fig S11D). *Eef2* was found to be annotated as an SVG by scGCO, SPARK-X, SpatialDE and Squidpy. This indicates genes with high levels of expression, but that do not display strong spatial expression patterns, may be a source of confounding.”

To further investigate the sensitivity and specificity of each of the analysis packages, two simulated datasets with known SVGs were generated using the SRTsim package. SRTsim allows the user to generate reference-free, simulate data that captures many of the features typical of Visium datasets. Further details on how these datasets were generate are available in the Methods section and are included below.

“Simulated datasets with known patterns of SVGs were generated using SRTsim (10). SRTsim allows the user to generate reference-free, simulated data that captures many of the features typical of Visium datasets through a Shiny app interface (10). These features include the number of spots and genes in the dataset, the mean and dispersion values of gene expression as well as the proportion of zero counts (10). If regions are selected within the dataset, the log fold-change within this region can be set compared to the rest of simulated area. Expression counts were simulated using a negative binomial distribution for both datasets (10). Two datasets were generated using SRTsim for this purpose. A square grid is selected (reproducible seed 2), with 4,000 spots and 15,000 genes overall. A square ‘hotspot’ pattern of 675 spots was generated in the centre of the grid, which will serve the area of higher gene expression for the simulated SVGs (Additional File 1: Fig S12A). 1,500 signal genes were then simulated with 13,500 noise genes, no lower signal genes were selected. A reproducible seed of 2 was set and baseline overdispersion was set to 0.5 with the baseline mean was set to 3. A second simulated dataset was generated with SRTsim with a slightly more complex gene expression pattern. The grid layout and number of spots was the same as the aforementioned dataset, set with reproducible seed 3. Here two separate regions were generated to contain simulated SVG expression, in opposite corners of the square layout (Additional File 1: Fig S12B). The two groups had a log fold-change of 5 compared to the rest of the area (Group A) with a log fold-change of 1. Next the expression of 15,000 genes were simulated across the dataset: 13,500 noise genes, 750 high signal SVGs and 750 low signal SVGs. Dispersion and mean values were the same as the previous dataset. This second dataset was generated with the aim of showcasing which packages may be adept at identifying SVGs with a lower signal across the dataset or a pattern different to the typical ‘hotspot’ which is often shown, such as in early simulations available with SPARK-X (2). The overlap of the results from each package with the known SVGs and noise genes in the data was then calculated to determine the sensitivity and specificity of each package. R scripts to calculate the sensitivity and specificity of each package were curated from code originally generated by ChatGPT (9).”

The SVG patterns generated for each dataset (Additional File 15: A-B) and the upset plots comparing the results from the analysis of these simulated datasets to the known SVGs are available in Additional File 1: Fig S12 C-D and included below:

Fig S12. Simulated datasets generated with SRT sim. A) Location of simulated SVGs with a hotspot pattern visualised in blue, while red area indicates expression of noise genes. B) Location of simulated SVGs in both blue and green corners, while red area indicates expression of noise genes. C) Distinct overlap of SVGs compared to the control SVG list identified by different combinations of the four tested packages. 1500 SVGs were present in this dataset. D) Distinct overlap of SVGs compared to the control SVG list identified by different combinations of the four tested packages. 750 high signal SVGs and 750 low signal SVGs were present in this dataset.

The sensitivity and specificity values of these analyses are provided in Additional file 2: Table S2.

Further discussion is available in the revised manuscript lines 75-85 and is included below.

“To gain insight into the inconsistent results between different packages we evaluated their sensitivity and specificity. To determine true positives and false positives rates, we first generated two simulated datasets with different known SVG patterns with SRTsim (Additional file 1: Fig. S12 A-B) (38). Each dataset contained 1500 known SVGs (see Methods). Using each as the input to four packages (scGCO, SpatialDE, Squidpy and SPARK-X), all packages identify all true positives across datasets, except for Squidpy when analysing the dataset with lower signal SVGs. This indicates a potential need for a more relaxed Moran’s I statistic threshold (Additional file 2: Table S2). Additionally, most packages introduce minimal levels of FPs with simulated datasets, except for scGCO (Additional file 2: Table S2). This is possibly due to of the type I error correction method or sequential search procedure employed by scGCO (31).”

The previously included negative simulated datasets were also used as inputs for the new included Squidpy analysis and the update results are available in lines 96-102 in the revised manuscript and below:

“For all negative control datasets generated from the FF left ventricle, Seurat, scGCO, Squidpy and SPARK-X identified no SVGs, indicating an ability to distinguish signal from noise. SpaGCN identified <10 SVGs. SpatialDE identified 4 SVGs when both spatial coordinates and columns of the gene expression matrix are permutated and 2953 in the other dataset. For negative control datasets generated from FFPE prostate randomising only spatial coordinates, SpatialDE identified 632 SVGs, SpaGCN identified 232 and Squidpy identified 1.”

**Comment 5: Among the 8 datasets tested, how does the performance change relative to the complexity of the tissue sections? One would expect that more complex tissue, like cancer would have more SVG? I suggest discussing more about this in relation to the datasets tested.**

**Response 5:** We investigated the complexity of tissues across biological conditions and the effect this might have on annotated SVGs across datasets. The new figure is included below and is available as Additional file 1: Fig S3.

Fig S3. Comparison of the number of SVGs identified by six different packages across different datasets. FFPE tissues are visualised by a cross and FF tissues are visualised with a square. High numbers of SVGs are identified in both FF and FFPE tissues, however tissues that are more transcriptionally complex seem to have more SVGs called across the dataset. Seurat reports a consistent number of SVGs across datasets as it first identifies highly variable genes then ranks their expression by how dependent it is on spatial location (52).

**Comment 6: It would be helpful to provide some information on best practice, for example a decision tree on in which case and/or for which datasets, users should use which method.**

**Response 6:** We thank the reviewer for this excellent suggestion and wish we could provide a clear-cut decision tree to select a method according to the input dataset. Indeed, we have investigated different cases of FF and FFPE tissue preservation and show that there is no preferred package that performed optimally for either case. This is displayed in Additional file 1: Fig S5 and see also response 5.

The discrepancies that we see between packages are consistent in FF and FFPE tissue. While we only explored a single platform for data generation, and further cross-platform comparisons could reveal which SVG calling methods perform best with data from a particular platform. The plot above also displays that packages tend to call more SVGs across tissues that are more transcriptionally complex overall, however the performance of a single package does not appear to be altered based on whether the input dataset is healthy or cancerous.

However, we can recommend:

- That method selection should not be performed based on whether the dataset comes from an FF or FFPE tissue samples as we’ve shown the discrepancies between packages are not based on factor.
- We have investigated also healthy and cancerous datasets and note that while more SVGs are called in cancerous datasets, this is in line with what one would expect from more transcriptionally heterogenous tissues. While a particular package does not improve performance in calling SVGs in this instance, inflated results may be observed, therefore users are cautioned to use more conservative FDR cut-offs, such as FDR < 0.01 rather than 0.05.
- As the SVGs identified by multiple packages appear to be *bona fide* SVGs, as displayed in Figure 2 and Additional file 14, we recommend the use of orthogonal methods when analysing tissues.
- When analysing tissues with more complex patterns with Squidpy, a less strict threshold for the Moran’s I statistic should be employed. This is because Squidpy was one of the few packages to report false negatives when analysing the SRTsim simulated dataset with lower signal SVGs.

These recommendations have been articulated in lines 141-160 of the revised manuscript and are included below:

“However, more transcriptionally heterogenous tissues appear to affect the performance of certain packages in calling SVGs and genes with unclear spatial patterns but high expression levels may be labelled as FP (14,15). Only four of the tested packages produced q-value ranked gene lists, thus differences in FP rates could confound comparisons of results across packages. Furthermore, due to the resolution of the Visium technology platform the presence of heterogenous cell types could complicate accurate identification of SVGs due to inherent transcriptional variation. Increased resolution may present challenges in terms of computational performance and the mathematical difficulties of dealing with highly sparse datasets. As SVG identification becomes an integral part of the computational analysis of SRT and datasets grow, a subset of SVGs could be used in preference over the entire dataset for downstream analysis (such as highly variable genes in scRNA-Seq analysis). Introducing noise may hamper key downstream steps in understanding spatially restricted disease states as well as novel targets for treatment (49,50). Methods that explore the entire dataset for SVG labelling paired with stricter q-value cut-offs (q-value < 0.01) can be employed when analysing tissues with known transcriptional complexity to decrease the possibility of FP being reported. Future work will include comparison of package performance across datasets generated from different SRT platforms. The development of workflows for simulating datasets and benchmarking current and novel methods for SVG identification will allow for accurate determination of the spatially restricted transcriptional differences that manifest as biological outcomes during development and disease.”

**Minor comments:**

**Comment 7: "The goal of SRT analysis pipelines is the robust and reliable identification of spatially variable gee (SVGs) within tissue sections (12,13)". I think there are many other goals. Please revise this sentence.**

**Response 7:** This has now been amended to the following in the revised manuscript:

"Among the goals of SRT analysis pipelines is the robust and reliable identification of spatially variable genes (SVGs) within tissue sections (12,13).”

**Comment 8: “We observed non overlapping parent terms between datasets in the top 10 over-represented GO terms (Fig. 1D)”-> should this be Fig 1B?**

**Response 8:** We thank the reviewer for pointing out this error, it has now been amended.

**Comment 9: Figure 1D show an example of a HVG gene. It is good to also include an example of a gene that is not spatially variable.**

**Response 9:** An example of a gene that is not spatially variable is visualised in Additional file 1: Fig S11 and is included below.

Fig S11. Spatial expression patterns of SVGs identified by all packages across the FF mouse brain coronal section dataset. A) Expression of *Hap1* across the hypothalamus and amygdala, cross-referenced with the Allen Mouse Brain Reference. B) Expression of *Prkcd* localised to the thalamus, cross-referenced with the Allen Mouse Brain Reference. C) Expression of *Itpka*, with highest expression in the isocortex, hippocampal formation (HPF) and cortical subplate consistent with patterns displayed in the Allen Mouse Brain Reference. D) Expression of *Eef2*, a known housekeeping gene (39).

**Reviewer #2:**

**The manuscript by Charitakis et al. describes a benchmarking analysis across five different software for analysis of spatially resolved transcriptomics datasets: SpatialDE, SPARK-X, Seurat, SpaGCN and scGCO. Specifically, the authors investigate the overlap across spatially variable genes (SVGs) identified by the five methods and find that they do not provide a consistent overlap among the five of them if not for genes with a stronger signal to noise ratio. In addition, the authors validate their results by using two negative control datasets obtained by permutation of spatial coordinates and gene expression matrix. From this analysis, Seurat, scGCO and SPARK-X identified no SVGs, indicating an ability to distinguish signal from noise, which is highly encouraging. Finally, the authors leverage two types of datasets: fresh frozen (FF) and formalin-fixed paraffin embedded (FFPE) tissues.** **This work is timely. With the increasing amount of spatial datasets available, it is highly valuable to identify appropriate tools for spatial analyses. However, I do have some major concerns:**

**Comment 10: SpatialDE is a relative old software (2018) which is not maintained to my knowledge. It is also the software that gives the most discrepancies. I recommend the authors to benchmark more recent software like Squidpy.** –

**Response 10:** We thank the reviewer for their comment and have added Squidpy to the list of packages that are used for analysis in this paper (Additional file 2: Table S3). We have applied Squidpy to all nine public datasets, and newly generated simulated data. We confirm that the SVGs predicted by Squidpy are statistically significantly different from those reported by other packages using a Wilcoxon signed rank test, further proving the disparities between the software (Additional file 2: Table S3). All of the analysis performed for the results of the original five software was re-run for the results from Squidpy.

Results from the analysis of public datasets visualised as upset plots are included in Figure 1A, included below, and in Additional files 2-4,9-11 and 13 for all nine other public datasets.

**Figure 1.** **Discrepancies between SVGs in a dataset annotated by six different packages.** **A)** Upset plots displaying the distinct intersections of SVGs identified in FF endometrial adenocarcinoma ovarian tissue dataset when analysed with SpaGCN, Squidpy, scGCO, Seurat, SPARK-X and SpatialDE. **B)** Pattern of expression of *JCHAIN*, a SVG identified by all six packages in FF endometrial adenocarcinoma ovarian tissue. Spots representing the capture regions of the Visium slide are overlayed on accompanying histological image. **C)** Sorted q-values from SpatialDE results across the FF left ventricle datasets plotted against SPARK-X q-values for the same gene. **D)** Gene ontology enrichment results from FF endometrial adenocarcinoma ovarian tissue dataset using SVGs identified by each package as inputs.

**Comment 11: While the authors reach the point of performing GO analysis of the SVGs to investigate ontology overlap across software, more thorough analysis should be carried out by investigating the spatial distribution of the SVGs deriving from the different software.**

**Response 11:** The spatial distributions of SVGs deriving from different software has been explored through the generation of two simulated datasets with known SVGs by SRTsim. Each dataset had SVGs that displayed distinct patterns from one another, visualised in Additional file 15: A-B, see also response 3.

The performance of each of the packages in identifying SVGs displaying these distinct patterns is outlined by the sensitivity and specificity of each package reported in Additional File 2: Table S2, please refer to response 3.

Squidpy seemingly has the most difficulty detecting SVGs that do not display a hotspot pattern (Additional File 18: Table S2), outlined in the revised manuscript in lines 79-82 included below:

“Using each as the input to four packages (scGCO, SpatialDE, Squidpy and SPARK-X), all packages identify all true positives across datasets, except for Squidpy when analysing the dataset with lower signal SVGs. This indicates a potential need for a more relaxed Moran’s I statistic threshold (Additional file 12: Table S2)”

**Comment 12: It would be helpful for general readers to have a brief summary of the differences in identifying SVGs across the five benchmarked methods.**

**Response 12:** The six packages chosen for this study were selected to demonstrate how different mathematical models are suited to labelling SVGs within Visium datasets. We’ve added a section discussing the differences in more detail in the Methods, lines 10-43) and included below:

"Six state of the art packages built for the identification of SVGs (Seurat and Squidpy excepted) in SRT data were selected for benchmarking: SpatialDE, SPARK-X, Seurat, SpaGCN, Squidpy and scGCO. Each package uses a different algorithm to identify SVGs and holds varied assumptions on the distribution of gene expression data (Additional File 2: Table S3). Packages were purposefully chosen to compass a variety of algorithms and associated mathematical models of gene distribution expression that can be used to identify these spatial patterns. While two packages used algorithms based on graph theory, the others were selected to test different mathematical assumptions regarding SRT data. SpatialDE employs Gaussian process regression, a non-parametric probabilistic model (Additional File 2: Table S3) (1). SPARK-X is another non-parametric method, building on a covariance test framework, specifically the projection covariance function ß(2). This function can measure similarity between two locations or gene expression, it can quantify if the product of the two inputs deviates significantly from the mean value (2). Seurat employs a mark point process, first used by the Trendsceek package (3,4). This is another non-parametric approach that can test if gene expression levels are significantly dependent on the spatial distribution of spots as a function of the distance between them (3). This can then calculate the mark-variogram (3,4). SpaGCN’s method is built around a graph convolutional network (GCN)-based approach and is unique in that it incorporates signal from histology images and restricts identification of SVGs to within spatial domains (5). Gene expression and image data are converted into a weighted undirected graph, which then calculates the distances between any two vertices in the graph (5). The nodes represent spots, and the edge weight is calculated using the histology image and the Euclidian distance between two vertices (5). The weight of each edge is calculated as a function between how related spots are in the graph (5). Dimensionality reduction then graph convolution is computed to then identify spatial domains (5). Finally, differential expression analysis is performed between spots in one spatial domain with neighbouring domains utilising a Wilcoxon rank-sum test and genes with an adjust p-value < 0.05 are reported as SVGs (5). scGCO is another graph-based method, but this package utilises a probabilistic graph model by optimising hidden Markov random fields (HMRF) for the purpose of SVG identification (6). Similar to Seurat, scGCO uses a marked point process to model spatial gene expression (4,6). The dependency of gene expression states on spatial locations is analysed using the complete spatial randomness framework, and scGCO overcomes its limitations using HRMF (6). Finally, Squidpy employs the spatial autocorrelation metrics of Moran’s I to label SVGs (7). Given a continuous feature, in this case gene expression level and their spatial location it can evaluate whether a pattern is present or not using Moran’s I (7).”

**Comment 13: Some extra considerations between FF and FFPE material to calculate SVGs would be appropriate.**

**Response 13:** We investigated all criteria and no notable differences have been identified in calling SVGs between FF and FFPE material, this is visualised in Additional file 1: Fig S3 , see above in response 5.

A description of the results is expanded on in the revised manuscript on lines 40-41:

“There is no apparent difference in reported SVGs driven by data generated from FF or FFPE tissue (Additional File 5).”

**Minor concern:**

**Comment 14: line 25: FF should stand for fresh frozen instead of formal fixed.**

**Response 14:** This has now been edited in line 45 of the revised manuscript.

**Second round of review**

**Reviewer 1**

The authors have addressed my comments.

A very minor suggestion is that the X-Axis and Y-Axis for the Figures 1A and 2A-D would not need the labels as Spatial1 and Spatial2 because these are just relative tissue location coordinates. It looks like the X:Y image ratio is not preserved (e.g. Figure 2A), but this information is not essential to understand the results.

I suggest acceptance of this interesting manuscript. Congratulations to all authors.

**Reviewer 2**

The authors have been responsive to the Reviewer's comments by expanding the tools for benchmarking (Squidpy) and adding explanatory text as suggested. I do not have further comments and recommend the article for publication in Genome Biology.
